# Spastin is an essential regulator of male meiosis, acrosome formation, manchette structure and nuclear integrity

**DOI:** 10.1242/dev.201183

**Published:** 2023-03-30

**Authors:** Samuel R. Cheers, Anne E. O'Connor, Travis K. Johnson, D. Jo Merriner, Moira K. O'Bryan, Jessica E. M. Dunleavy

**Affiliations:** ^1^ School of BioSciences and Bio21 Institute, The University of Melbourne, Parkville, VIC 3010, Australia; ^2^ School of Biological Sciences, Monash University, Clayton, VIC 3800, Australia

**Keywords:** Male infertility, Spermatogenesis, Microtubule severing, AAA ATPase, Hereditary spastic paraplegia

## Abstract

The development and function of male gametes is dependent on a dynamic microtubule network, yet how this is regulated remains poorly understood. We have recently shown that microtubule severing, via the action of the meiotic AAA ATPase protein clade, plays a crucial role in this process. Here, we sought to elucidate the roles of spastin, an as-yet-unexplored member of this clade in spermatogenesis. Using a *Spast^KO/KO^* mouse model, we reveal that spastin loss resulted in a complete loss of functional germ cells. Spastin plays a crucial role in the assembly and function of the male meiotic spindle. Consistent with meiotic failure, round spermatid nuclei were enlarged, indicating aneuploidy, but were still able to enter spermiogenesis. During spermiogenesis, we observed extreme abnormalities in manchette structure, acrosome biogenesis and, commonly, a catastrophic loss of nuclear integrity. This work defines an essential role for spastin in regulating microtubule dynamics during spermatogenesis, and is of potential relevance to individuals carrying spastin variants and to the medically assisted reproductive technology industry.

## Teaser

**Summary:** We identify an essential role for the microtubule-severing enzyme spastin in the regulation of microtubule dynamics during spermatogenesis in mouse.

## INTRODUCTION

Microtubule severing is fundamental to the regulation of microtubule dynamics and is achieved via members of the meiotic clade of the ‘ATPases associated with diverse cellular activities’ (AAA) superfamily. This group includes the katanins, the fidgetins and spastin (SPAST), which all have microtubule-severing activity, in addition to VPS4, which has no known severing function (Frickey and Lupas, 2004). Although the function of microtubule severing in most mammalian developmental processes is unexplored, crucial roles in neurodevelopment are well established for spastin and the katanins (Ahmad et al., 1999; Chen et al., 2014; Hu et al., 2014; Liu et al., 2021; Tan et al., 2020; Yu et al., 2008), and in multiple aspects of male germ cell development for the katanins (Dunleavy et al., 2021, 2017; O'Donnell et al., 2012). *SPAST* mutations are the most common cause of hereditary spastic paraplegia (Hazan et al., 1999). Hereditary spastic paraplegia is characterised by progressive degeneration of neurons in the central nervous system, resulting in lower limb stiffness, weakness and spasticity. The *SPAST* mutation is dominant; however, it is still debated whether disease is caused by haploinsufficiency or by gain of function, although it appears that an interplay of both mechanisms is likely (Qiang et al., 2019). Although spastin is expressed in other tissues, its roles outside the nervous system remain virtually unexplored. Of direct relevance to this study, spastin is highly expressed in male germ cells.

To sever microtubules, ATP-bound spastin subunits assemble into a spiral-shaped homohexamer around the C-terminal tail of tubulin (Roll-Mecak and Vale, 2008; Sandate et al., 2019). Upon hydrolysis of ATP, the hexamer changes conformation to a ring and in this movement tugs upon the tubulin C-terminal tail, removing the tubulin heterodimer from the microtubule lattice (Roll-Mecak and Vale, 2008; Zehr et al., 2020). The action of spastin and other microtubule-severing enzymes can lead to microtubule disassembly, the release of microtubules from nucleation sites and the generation of short stable seeds of microtubules for transport to other parts of the cell and/or to nucleate microtubule growth (reviewed by McNally and Roll-Mecak, 2018). Conversely, and perhaps counterintuitively, spastin action can lead to microtubule stabilisation by removing GDP-associated tubulin heterodimers from the microtubule lattice, which are then replaced with more stable GTP-associated tubulin heterodimers (Vemu et al., 2018).

In addition, through the MIT domain, spastin is also able to interact with components of the ESCRT-III machinery (Reid et al., 2005; Yang et al., 2008). Through these interactions, spastin has been shown to be involved in endosome formation and processing, nuclear envelope reformation, and midbody abscission during cytokinesis (reviewed by Migliano et al., 2022). Specifically, ESCRT-III has been shown to recruit spastin to the midbody during mitotic cytokinesis in HeLa cells to sever the midbody microtubules and allow the completion of membrane fission (Connell et al., 2009). Similarly, after cell division in HeLa cells, ESCRT-III recruits spastin to sites on the reforming nuclear membrane through which microtubules pass. Severing of these microtubules allows for sealing of the nuclear membrane (Vietri et al., 2015). Spastin is also required in HeLa cells and mouse embryonic fibroblasts for endosomal tubulation and fission, and correct lysosome function (Allison et al., 2017, 2013). Both of these functions require interaction with ESCRT-III components and the ability of spastin to sever microtubules. Finally, spastin is involved in the movement and metabolism of lipid droplets in HeLa cells, which, interestingly, requires interaction with ESCRT-III components but not microtubule severing (Arribat et al., 2020; Chang et al., 2019). Given the central roles of cell division, nuclear remodelling and lipid and vesicle movement in spermatogenesis, the possibility exists that spastin plays a role in male fertility beyond the regulation of microtubule bulk.

The development of male germ cells, like neurogenesis, is highly dependent on complex microtubule structures. These include the bipolar spindle and midbody during mitosis and meiosis, the manchette for sperm head shaping, and the axoneme, which forms the architectural core of the sperm tail. Previous research has shown that spermatogenesis is dependent on microtubule severing through other members of the meiotic group of the AAA superfamily, the katanins KATNB1, KATNA1, KATNAL2 (summarised by Dunleavy et al., 2022 preprint). The role of spastin in the development of male germ cells has not yet been directly tested. However, spastin is highly expressed in the testis (Karlsson et al., 2021), and, notably, a previous publication reported that homozygous spastin mutant mice were male sterile, but the biological origin of this phenotype was not investigated (Tarrade et al., 2006).

Here, we directly tested the role of spastin in spermatogenesis using a whole-body *Spast* knockout mouse model in which a truncation occurred after exon 4. We reveal that spastin is essential for male germ cell development in the mouse and loss of spastin is incompatible with the production of male germ cells and male fertility. Our work identifies spastin as a regulator of anaphase during meiosis, of acrosome biogenesis and of the sculpting of the sperm head via the manchette. Interestingly, we also find that spastin plays a crucial role in maintaining haploid germ cell nuclear integrity. Finally, using proteomics approaches we define the spastin testis interactome, which includes key microtubule-, actin- and septin-related proteins. Collectively, this work defines multiple distinct roles for spastin in spermatogenesis and identifies spastin as an essential component of the testis microtubule-severing toolbox wherein multiple enzymes are required, each of which have unique and multifaceted roles.

## RESULTS

### Spastin is required for spermatogenesis and male fertility

**Fig. 1.**
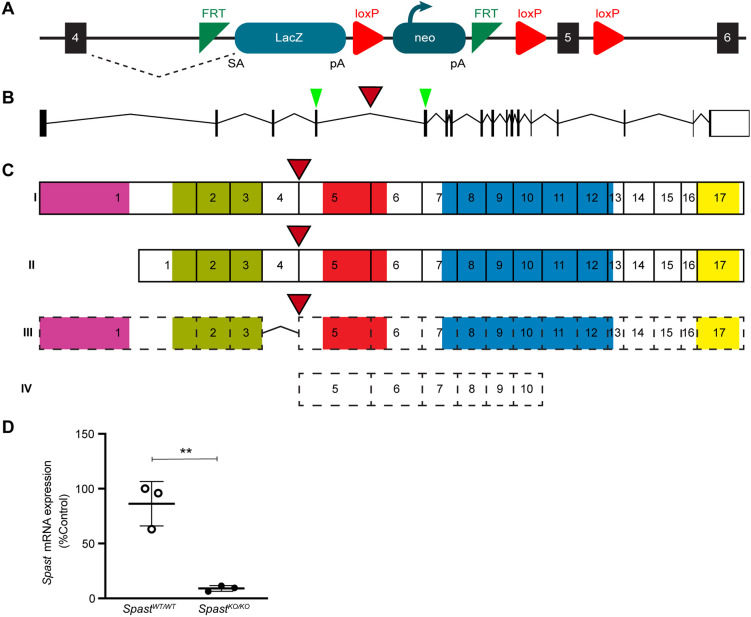
**Ablation of spastin function in *Spast^KO/KO^* mice.** (A) The *Spast* KO-first conditional ready allele. The FRT-lacZ-loxP-neo-FRT-loxP-*Spast* exon 5-loxP cassette was inserted into *Spast* intron 4. The dashed line represents alternative splicing from exon 4 to the beginning of the inserted cassette. pA, polyA signal; SA, splice acceptor. (B,C) Schematics of the murine *Spast* gene (B) and SPAST protein (C). The red arrowhead indicates the point of cassette insertion. The green arrowheads indicate the target regions of the qPCR primers. The hydrophobic region, MIT domain, MTBD domain, AAA ATPase domain, and VPS4 oligomerisation domain are shown in pink, green, red, blue and yellow, respectively. Two *Spast* isoforms M1 (I) and M87 (II) have been characterised in mice (Mancuso and Rugarli, 2008). Two additional *Spast* isoforms (III, IV), which are yet to be validated, are predicted (UniProt, A0A286YCJ4 and A0A3B2WBA7). (D) qPCR analysis of *Spast* transcript levels in *Spast^WT/WT^* and *Spast^KO/KO^* whole testes (*n*=3 mice/genotype). Data are shown as mean±s.d. and are normalised to the expression of *Ppia*. **P=0.0028 (unpaired Student's *t-*test).

To investigate the role of spastin in male germ cell development, we used a whole-animal *Spast* knockout mouse model (*Spast^KO/KO^*) comprising a trans-NIH Knockout Mouse Project (KOMP) construct (Fig. 1A) inserted into *Spast* intron 4 (Fig. 1B,C, red arrowhead) designed to truncate *Spast* mRNA at exon 4. The homozygous presence of the construct resulted in an 89.5% reduction in *Spast* mRNA expression in *Spast^KO/KO^* compared with wild-type (*Spast^WT/WT^*) testes (Fig. 1D). Sequencing of the PCR product confirmed that low levels of *Spast* mRNA-containing sequences from the construct were produced in the *Spast^KO/KO^* mouse. This indicates that, in common with several other KOMP constructs, a low degree of transcription occurred. Owing to the presence of the construct in the mRNA, it is unlikely that translation would result in functional spastin. The loss of spastin at a protein level was confirmed by the immunolabeling of testis sections (Fig. S1A,B). This is the first study to use this construct as a whole-body knockout. Previous studies using this mouse model generated a tissue-specific conditional knockout (Brill et al., 2016; Magiera et al., 2018).

**Fig. 2.**
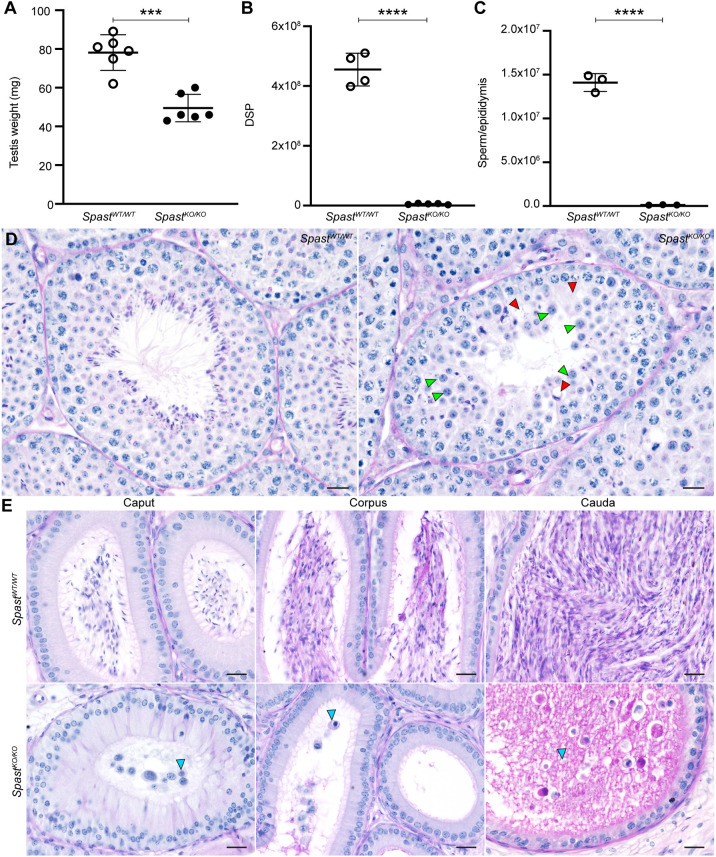
**Spermatogenic defects due to knockout of *Spast*.** (A-C) Testis weight (A), total daily sperm production (DSP) per testis (B) and epididymal sperm content (C) in *Spast^KO/KO^* mice (black circles) compared with *Spast^WT/WT^* controls (white circles) (*n*≥3 mice/genotype, mean±s.d.). ***P=0.0001, ****P<0.0001. (D) PAS-stained testis sections from *Spast^WT/WT^* and *Spast^KO/KO^* mice. Red arrowheads indicate vacuoles in the seminiferous epithelium. Green arrowheads indicate abnormally large round spermatids. Scale bars: 20 µm. (E) Epididymis sections from *Spast^WT/WT^* and *Spast^KO/KO^* mice. Blue arrowheads indicate prematurely released germ cells. Scale bars: 20 µm.

*Spast^KO/KO^* mice generated from the intercrossing of heterozygous mice were born at the expected Mendelian frequency and did not show any overt abnormalities. *Spast^KO/KO^* male mice exhibited normal mating behaviour when partnered with *Spast^WT/WT^* female mice; however, they were uniformly male sterile (8.5 pups per copulatory plug in *Spast^WT/WT^*; *n*=3 males, 2 copulatory plugs per male) versus 0.0 pups in *Spast^KO/KO^* (*n*=4 males, 2 copulatory plugs per male; *P*≤0.0001). *Spast^WT/KO^* males and *Spast^KO/KO^* females produced litters of normal size and so were not explored further. Analysis of the *Spast^KO/KO^* male reproductive tract revealed the complete absence of sperm. *Spast^KO/KO^* mice had normal body weight, but significantly smaller adult testes compared with *Spast^WT/WT^* controls (36.7% reduction; Fig. 2A). An analysis of testis daily sperm production revealed that *Spast^KO/KO^* mice produced 99.4% fewer sperm (Fig. 2B), and their epididymal sperm content was reduced by 99.7%, compared with *Spast^WT/WT^* controls (Fig. 2C). Rare cells that were seen in the epididymis of *Spast^KO/KO^* mice were prematurely sloughed spermatocytes and round spermatids rather than spermatozoa (Fig. 2D,E, blue arrowheads). This was in stark contrast to *Spast^WT/WT^* epididymides, which were full of spermatozoa (Fig. 2E, cauda).

**Fig. 3.**
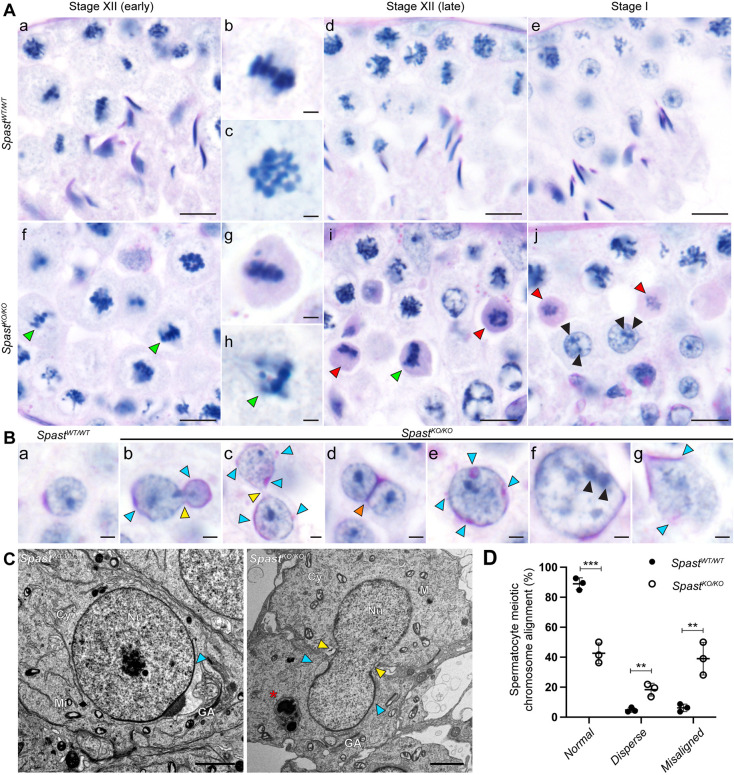
**Spastin is essential for correct meiotic division.** (A) PAS-stained testis sections from *Spast^KO/KO^* mice had an increase in pyknotic spermatocytes (red arrows) in stage XII and I tubules. Green arrowheads indicate wide dispersion or misalignment of chromosomes. Black arrowheads indicate multiple nucleoli within abnormally large nuclei. Panels b,c,g,h show meiotic cells at increased magnification. Scale bars: 10 µm (a,d-f,i,j); 2 µm (b,c,g,h). (B) The meiosis abnormalities resulted in round spermatids with abnormal phenotypes, including sister cells sharing a single nucleus, which crossed the intercellular bridge (yellow arrowheads), binucleated spermatids (orange arrowhead), and abnormally large nuclei (e-g). Multiple, or fragmented, acrosomes are also indicated by blue arrowheads. Scale bars: 2 µm. (C) Transmission electron microscopy showing failure of meiosis resulting in a nucleus crossing the intercellular bridge between two daughter cells. The position of the intercellular bridge is indicated by yellow arrowheads; the position of the acrosome is indicated by blue arrowheads. Asterisk indicates what is likely to be a late-stage multilamellar body, which may have formed as a result of an overactive Golgi-apparatus or the over-activation of phagocytic pathways. This structure was frequently observed in *Spast^KO/KO^* mice, but was not observed in *Spast^WT/WT^* mice. Cy, cytoplasm; GA, Golgi apparatus; Mi, mitochondrion; Nu, nucleus. Scale bars: 2 µm. (D) Quantification of common phenotypic defects seen in meiosis in PAS-stained testis sections of *Spast^KO/KO^* mice (black circles) compared with *Spast^WT/WT^* controls (white circles) (*n*=3/genotype; for each animal, all metaphase spermatocytes in five stage XII seminiferous tubules were assessed). For graphed data, lines represent mean±s.d. **P<0.01, ***P<0.001 (unpaired Student's *t-*test).

Histological analysis of *Spast^KO/KO^* testes identified multiple abnormalities at various stages of spermatogenesis. Consistent with premature germ cell sloughing and/or death, large areas of the seminiferous epithelium were devoid of germ cells and/or exhibited a ‘lacy’ appearance in *Spast^KO/KO^* testes, indicative of recent germ cell loss (Fig. 2D, red arrowheads). In the majority of *Spast^KO/KO^* seminiferous tubules, spermatogonia and primary spermatocytes up to and including prophase I appeared phenotypically normal. The earliest point at which a clear defect could be seen in the *Spast^KO/KO^* mice was during metaphase of meiosis I. During meiotic division, cells often displayed misaligned chromosomes and signs of division failure, resulting in abnormally large round spermatids (Fig. 2D, green arrowheads) or, rarely, bi-nucleated spermatids (Fig. 3B, orange arrowhead), indicating that spastin may have a crucial role in meiosis. We also noticed that round spermatids in *Spast^KO/KO^* testes had abnormal acrosome development and failed to elongate, indicating an essential role for spastin during the early processes of spermiogenesis.

Consistent with spastin regulating multiple phases of germ cell development, assessment of spastin localisation in purified wild-type mouse germ cells revealed that spastin was present in the cytoplasm of male germ cells throughout spermatogenesis (Fig. S1C). Notably, it colocalised with cytoplasmic microtubules in spermatocytes and spermatids, including the peri-acrosomal microtubules in round spermatids, and with the microtubule-based manchette in elongating spermatids (Fig. S1C). These findings are consistent with previous single-cell RNA sequencing data, which revealed that *Spast* is expressed throughout spermatogenesis in mice with expression peaking during meiosis and spermiogenesis (Fig. S1D; Ernst et al., 2019).

### Spastin is essential for meiotic spindle formation and function in male germ cells

Consistent with the significant reduction in sperm output, there was a significant 2.6-fold increase in the number of apoptotic germ cells per seminiferous tubule in *Spast^KO/KO^* mice compared with *Spast^WT/WT^* littermates (Fig. S2A). The increase in apoptosis occurred primarily in metaphase and early anaphase spermatocytes, supporting an essential role for spastin in male mammalian meiosis (Fig. S2B,C). Indeed, detailed analysis of *Spast^KO/KO^* periodic acid–Schiff (PAS)-stained testis sections revealed that metaphase spermatocytes frequently contained misaligned chromosomes (Fig. 3A, green arrowheads), and/or a wider dispersion of chromosomes at the metaphase plate (Fig. 3A, green arrowheads). These phenotypes were rarely observed in *Spast^WT/WT^* controls. On average, 39% of meiotic cells in *Spast^KO/KO^* mice contained misaligned chromosomes and 18% were abnormally dispersed. In contrast, on average, 6% of meiotic cells in *Spast^WT/WT^* mice were misaligned and 5% were abnormally dispersed (Fig. 3D). In the *Spast^KO/KO^* germ cells that progressed to anaphase uneven chromosome segregation was common (Fig. 3Bb).

In *Spast^WT/WT^* testis sections, meiosis I completed in stage XII tubules, as expected, and meiosis I or II spermatocytes were not seen in subsequent stage I tubules (Fig. 3A). In contrast, in the *Spast^KO/KO^* testis, many pyknotic PAS-positive/caspase-positive metaphase I and early anaphase I spermatocytes were observed to arrest development in stage XII and persisted in stage I tubules (Fig. 3A, red arrowheads, Fig. S2B,C) indicative of a meiosis arrest followed by germ cell loss. Of the *Spast^KO/KO^* germ cells that completed meiosis, many of the resultant round spermatids were abnormal. In *Spast^KO/KO^* males, round spermatids often had abnormally large nuclei (Fig. 3Be-g) containing multiple nucleoli, or the presence of multiple nuclei within the same cell. In contrast, round spermatids from *Spast^WT/WT^* males (Fig. 3Ba) had uniformly sized nuclei with a single nucleolus. The absence of a corresponding number of abnormally small diameter spermatids suggests a major failure of chromosome segregation involving the collapse of at least two sets of chromosomes into a single spermatid nucleus (Fig. 3B). An additional unusual phenotype we frequently observed in *Spast^KO/KO^* mice was a single nucleus crossing the intercellular bridge between sister round spermatids (Fig. 3B, yellow arrowheads). This phenotype was never seen in wild-type mice and is suggestive of increased malleability of mutant cells and a failure of metaphase/anaphase and incomplete cytokinesis. In addition, rare binucleated spermatids were observed in the *Spast^KO/KO^* mice (Fig. 3B, orange arrowhead). These cells likely arose as a result of complete anaphase followed by unsuccessful cytokinesis. Neither of these phenotypes was observed in the round spermatids from *Spast^WT/WT^* mice (Fig. 3Ba).

### Spastin is required for acrosome development

**Fig. 4.**
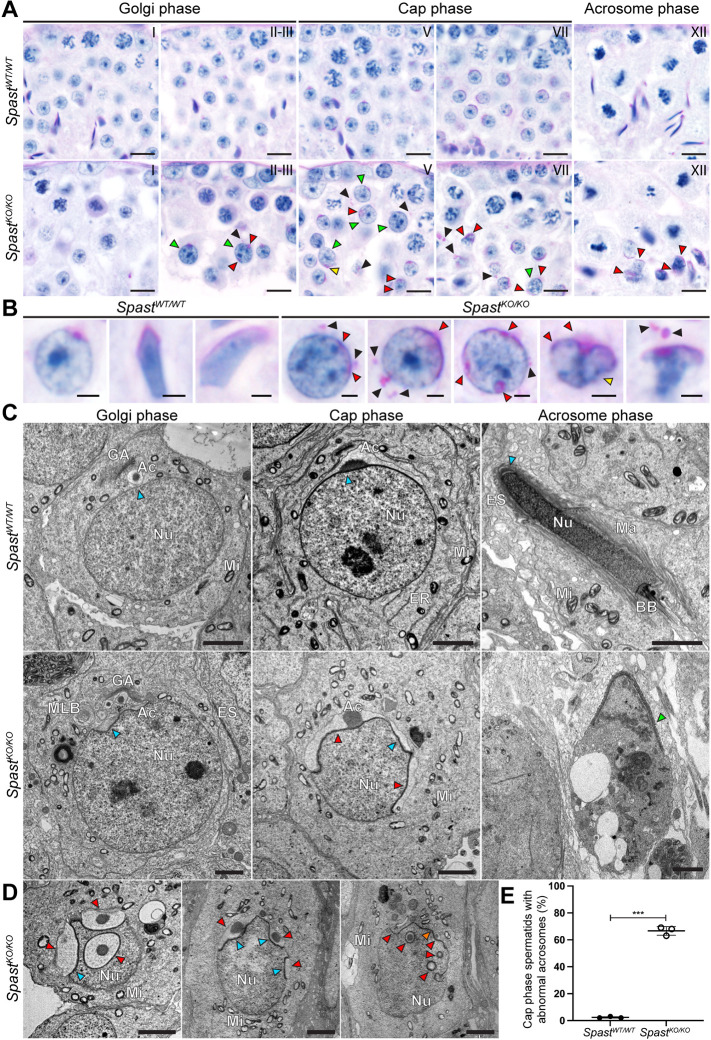
**Spastin is essential for formation of the acrosome.** (A) The absence of spastin resulted in multiple defects during acrosome development as observed in PAS-stained testis sections. Red arrowheads indicate the presence of multiple acrosomal vesicles at the nuclear surface, and black arrowheads indicate pro-acrosomal vesicles mislocalised throughout the cytoplasm. The yellow arrowhead indicates an acrosomal vesicle developing on a nucleus traversing the intercellular bridge, and green arrowheads indicate overtly abnormally large spermatid nuclei. Roman numerals indicate seminiferous tubule stage. Scale bars: 10 µm. (B) High-power images of spermatids from *Spast^WT/WT^* and *Spast^KO/KO^* males throughout acrosome development. Red arrowheads indicate the presence of multiple acrosomal vesicles at the nuclear surface, and black arrowheads indicate pro-acrosomal vesicles mislocalised throughout the cytoplasm. The yellow arrowhead indicates a binucleated spermatid. Scale bars: 2 µm. (C,D) Transmission electron microscopy showing the ultrastructure of the acrosome in spermatids from *Spast^WT/WT^* and *Spast^KO/KO^* males. In C, progressive steps of acrosome development are shown from left to right. The blue arrowheads indicate an abnormal invagination of the nuclear membrane below the developing acrosomal granules, and lack of this invagination in the *Spast^WT/WT^* mice. Red arrowheads indicate sites of supernumerary acrosome formation. Orange arrowhead indicates abnormal nuclear membrane morphology in the absence of the acrosomal vesicle. Green arrowhead indicates the location of a compacted nucleus. Ac, acrosome; BB, basal body; ER, endoplasmic reticulum; ES, ectoplasmic specialisation; GA, Golgi apparatus; Ma, manchette; Mi, mitochondria; MLB, multilamellar body; Nu, nucleus. Scale bars: 2 µm. (E) Percentage of cap-phase spermatids in *Spast^WT/WT^* and *Spast^KO/KO^* mice presenting with abnormal acrosome localisation and/or morphology in PAS-stained testis sections (*n*=3/genotype; for each animal, all cap-phase spermatids in five seminiferous tubules were assessed). For graphed data, lines represent the mean±s.d. ***P<0.001 (unpaired Student's *t-*test).

Despite the meiotic disruptions observed in *Spast^KO/KO^* testes, the processes that govern the morphogenesis of round spermatids into elongated spermatids continued in *Spast^KO/KO^* germ cells. One of the earliest morphological events is acrosome formation, which occurs at the apical surface of the sperm nucleus. This structure is required for the penetration of the cells surrounding the oocyte and thus fertilisation. It begins with the production of pro-acrosomal vesicles in step 2-3 spermatids. In wild-type spermatids, these vesicles are transported to the apical pole of the nucleus where they adhere to the nuclear envelope via the acroplaxome to form a single acrosomal vesicle (Fig. 4A). During the Golgi phase (step 2-3) of acrosome development, pro-acrosomal vesicles are solely derived from the Golgi, whereas in the cap phase of development (step 4-7) both Golgi- and endocytic pathway-derived vesicles progressively enlarge the acrosome as it flattens and spreads to cover the apical half of the nucleus (Fig. 4A, Cap phase) (Pleuger et al., 2020).

We observed that early round spermatids from *Spast^KO/KO^* males (step 2-3) had PAS-positive, pro-acrosomal vesicles that were ectopically distributed throughout the cytoplasm (Fig. 4A, black arrowheads). In many spermatids, pro-acrosomal vesicles were observed to adhere to multiple ectopic sites on the nuclear membrane, including at the caudal pole (Fig. 4A, red arrowheads; Fig. 4B-D), suggesting a disruption of the cytoskeletal network required for pro-acrosomal vesicle transport from the Golgi to the nuclear membrane. Moreover, as spermatids from *Spast^KO/KO^* males developed into cap phase, this resulted in supernumerary acrosomes (Fig. 4B-D, red arrowheads). Quantitative analysis of acrosome abnormalities in cap phase spermatids identified a significant increase in ectopic and abnormal acrosomes within *Spast^KO/KO^* mice (Fig. 4E). Additionally, multi-lamellar bodies were frequently observed in spermatids from *Spast^KO/KO^* mice from cap phase onwards (Fig. 3C, asterisk), indicating that the Golgi apparatus and/or the endocytic pathway may be overactive (Hariri et al., 2000).

In the acrosome phase of development (step 8-12 spermatids), the acrosome could be seen as a thin vesicle coating the entire anterior region of the sperm head in spermatids from *Spast^WT/WT^* males (Fig. 4C, Acrosome phase). A similar compacted acrosome phenotype was seen in the spermatids from *Spast^KO/KO^* males (Fig. 4C, green arrowhead); however, multiple acrosome compartments were still observed (Fig. 4C, red arrowheads), in addition to a loss of nuclear membrane integrity (Fig. 4C, Acrosome phase). Electron microscopy revealed that docking of acrosomal vesicles throughout development, starting in the Golgi phase, was associated with an abnormally deep nuclear membrane invagination in spermatids from *Spast^KO/KO^* males, suggesting compromised nuclear integrity (Fig. 4C,D, blue arrowheads).

### Spastin is required for the maintenance of nuclear membrane integrity

**Fig. 5.**
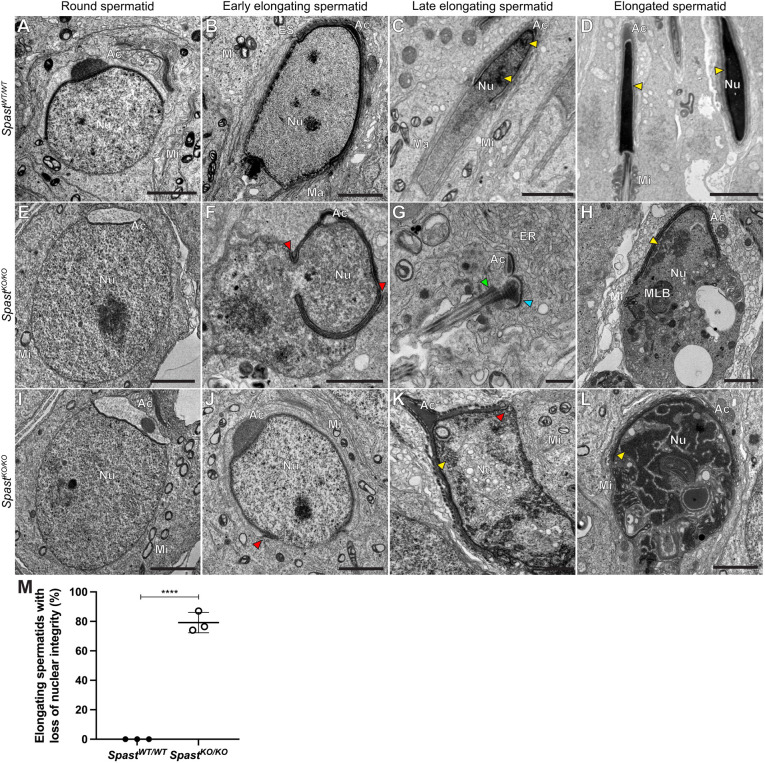
**Spastin is required for the maintenance of spermatid nuclear membrane integrity.** (A-L) Transmission electron microscopy of developing spermatids from *Spast^WT/WT^* and *Spast^KO/KO^* mice. In *Spast^KO/KO^* mice, following the initiation of spermatid elongation, spermatids presented with a loss of nuclear membrane integrity, ultimately resulting in cell death and a virtual absence of sperm. Red arrowheads indicate the site of membrane rupture. Blue arrowhead indicates the basal plate of the head-tail coupling apparatus. Yellow arrowheads indicate condensed DNA. Green arrowhead indicates the basal body. Ac, acrosome; ER, endoplasmic reticulum; ES, ectoplasmic specialisation; Ma, manchette; Mi, mitochondria; MLB, multilamellar body; Nu, nucleus. Scale bars: 2 µm (A-F,H-L); 500 nm (G). (M) Percentage of elongating spermatids in *Spast^WT/WT^* and *Spast^KO/KO^* males presenting with nuclear integrity defects in electron micrographs of seminiferous tubules (*n*=3/genotype; for each animal, a minimum of 48 elongating spermatids were assessed). The graphed data represent the mean±s.d. ****P<0.0001 (unpaired Student's *t-*test).

One of the more unusual manifestations of spastin loss was a severe disruption to spermatid nuclear integrity at the onset of nuclear elongation in step 9 (Fig. 5E-L). We identified a significant increase in the percentage of elongating spermatids that had a loss of nuclear integrity in *Spast^KO/KO^* mice (Fig. 5M). This phenotype was never observed in *Spast^WT/WT^* controls (Fig. 5A-D,M). Nuclear envelope breakages were first apparent in early elongating spermatids from *Spast^KO/KO^* mice (Fig. 5F,J). At later developmental stages, the nuclear envelopes of spermatids from *Spast^KO/KO^* males became increasingly degraded and the mixing of nuclear and cytoplasmic material continued until they were indistinguishable from each other (Fig. 5E,F,H-L). Ruptured nuclear membranes were most frequently observed at the caudal pole (Fig. 5J, red arrowhead), and were rarely seen at the acrosome-covered apical pole, possibly owing to a stabilising effect of the acrosome and/or acroplaxome on the membrane. Alternatively, the pressure applied by the manchette, which envelops the caudal half of the spermatid from step 8/9 onwards may be a trigger for rupture. As described by Lehti and Sironen (2016), the manchette is a transient microtubule-based structure that plays a pivotal role in sculpting the distal half of the spermatid nucleus.

We also noted that DNA condensation was disrupted in spermatids from *Spast^KO/KO^* mice. In elongated spermatids from *Spast^WT/WT^* mice, the nuclear material became progressively more electron dense as DNA condensed (Fig. 5, yellow arrowheads). In elongating spermatids from *Spast^KO/KO^* males, DNA condensation was only initiated in isolated regions (Fig. 5, yellow arrowheads). In the later steps of spermiogenesis, and in contrast to the situation in wild type, which was replete with elongating and elongated spermatids, these defects collectively resulted in most spermatids from *Spast^KO/KO^* males containing no discernible nucleus (Fig. 5K,L). However, fragments of the nuclear membrane associated with the acrosome (Fig. 5K) and/or the basal body from which the sperm tail initiates (Fig. 5G, blue arrowhead) were visible. Consistent with these defects, spermatids from *Spast^KO/KO^* males had an increase in DNA damage compared with *Spast^WT/WT^* as assessed by marking γ-H2AX, an indicator of double-stranded DNA breaks (Fig. S3).

### Spastin is required for manchette development and sperm head shaping

**Fig. 6.**
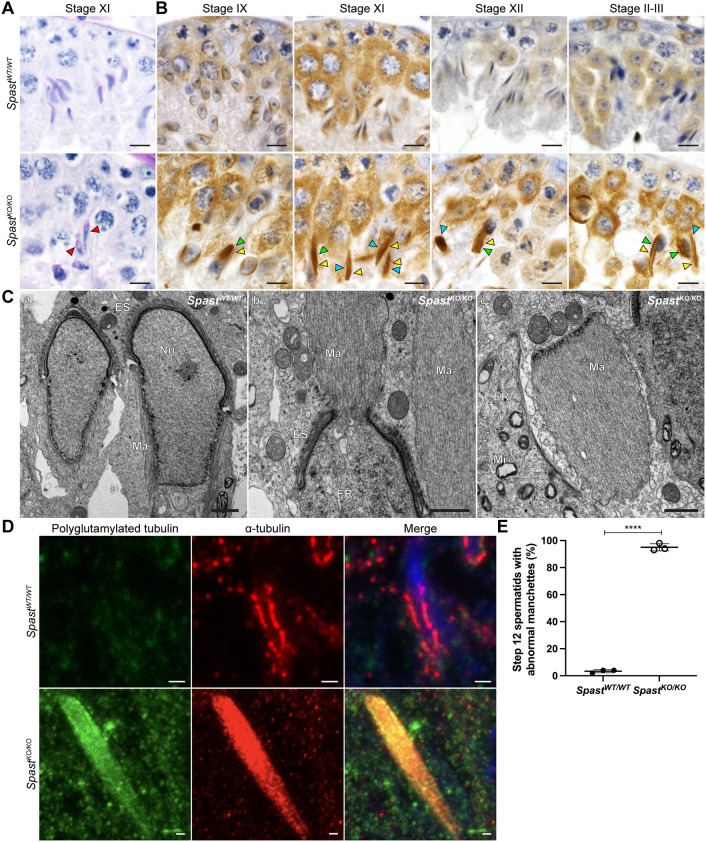
**Spastin is a key regulator of manchette structure and dynamics.** (A) PAS-stained testis sections showing normal elongating spermatids in *Spast^WT/WT^* mice and abnormal elongating spermatids in *Spast^KO/KO^* mouse testes (red arrowheads). Scale bars: 20 µm. (B) *Spast^WT/WT^* and *Spast^KO/KO^* testis sections immunolabelled for α-tubulin, a core component of microtubules within the manchette. The tubule stages that capture manchette formation, migration and disassembly are shown from left to right. Green and blue arrowheads, respectively, indicate manchettes that have partially or completely dissociated from the nucleus. Yellow arrowheads indicate manchettes of abnormal size. Scale bars: 20 µm. (C) TEM showing the manchette ultrastructure in *Spast^WT/WT^* and *Spast^KO/KO^* mice. In panel b, a manchette dissociating from the nucleus of a spermatid in a *Spast^KO/KO^* male can be observed, and in panel c a dissociated manchette is shown. ER, endoplasmic reticulum; ES, ectoplasmic specialisation; Ma, manchette; Mi mitochondria; Nu, nucleus. Scale bars:1 µm. (D) Immunostaining of manchettes showing an increase in microtubule number (red) and polyglutamylated tubulin (green) in *Spast^KO/KO^* compared with *Spast^WT/WT^* mice. Nuclei are counterstained with DAPI (blue). Staining for polyglutamylated tubulin identified an overall increase in polyglutamylated tubulin in *Spast^KO/KO^* spermatids, especially in the microtubules of the manchette, consistent with polyglutamylated tubulin being the preferred target for spastin-mediated microtubule severing. Scale bars: 1 µm. (E) Percentage of step 12 spermatids in *Spast^WT/WT^* and *Spast^KO/KO^* males presenting with abnormal manchettes in α-tubulin-stained testis sections (*n*=3/genotype; for each animal, all step 12 spermatids in five seminiferous tubules were assessed). The graphed data represent the mean±s.d. ****P<0.0001 (unpaired Student's *t-*test).

Spermatid head shaping is mediated in part by the manchette (reviewed by Dunleavy et al., 2019a), a transient structure made up of microtubules that extend caudally from a perinuclear ring immediately distal to the leading edge of the acrosome. In spermatids from *Spast^WT/WT^* males, the manchette forms at step 8, and as spermatogenesis progresses the manchette moves distally towards the centriole/basal body and the growing sperm tail (Fig. 6B). In parallel, the perinuclear ring constricts, thus acting to sculpt the distal half of the sperm head (Fig. 6B, Stage XI). Once sperm head shaping is complete, the manchette is disassembled in step 14 spermatids (Fig. 6B, Stage II-III).

In *Spast^KO/KO^* mice, the manchette (marked by α-tubulin) formed at the appropriate time, but was phenotypically abnormal (Fig. 6B). Manchette microtubules were observed to over-accumulate resulting in abnormally wide and dense manchettes, suggestive of a role for spastin in microtubule pruning (Fig. 6B, yellow arrowheads). Consistent with this interpretation, by step 11, manchettes in *Spast^KO/KO^* males were excessively long compared with those seen in *Spast^WT/WT^* males (Fig. 6B). Further, manchettes were still present in step 14 spermatids from *Spast^KO/KO^* (stage II-III), whereas in spermatids from *Spast^WT/WT^* males they were disassembled (Fig. 6A, Stage II-III), indicating that, like katanin proteins (Dunleavy et al., 2021, 2017), spastin influences the dissolution of the manchette. Quantitative analysis of step 12 spermatids confirmed there was a significant increase in the percentage with abnormal manchettes, including ectopic, abnormally dense and excessively elongated manchettes, in *Spast^KO/KO^* compared with *Spast^WT/WT^* mice (Fig. 6E). The absence of spastin resulted in the partial (Fig. 6B, green arrowheads; Fig. 6Cb) or complete detachment (Fig. 6B, blue arrowheads; Fig. 6Cc) of the manchette from most elongating spermatid nuclei. This dissociation could be due to the degradation of the nuclear membrane resulting from the compromised nuclear integrity explored above.

Previous studies have shown that spastin preferentially severs polyglutamylated tubulin and that knockout of spastin resulted in an increase of polyglutamylated microtubules (Lacroix et al., 2010; Magiera et al., 2018). As such, we hypothesised that if spastin were required to sever manchette microtubules, there would be an increase of polyglutamylated microtubules in the manchettes of *Spast^KO/KO^* testis sections. To test this, we marked testis sections for α-tubulin as a marker of the manchette, in addition to polyglutamylated tubulin. In *Spast^KO/KO^* mice, we found that, not only was manchette microtubule density increased compared with *Spast^WT/WT^*, but there was an increased accumulation of polyglutamylated microtubules (Fig. 6D). Collectively, these results strongly suggest that, within male germ cells, spastin severs polyglutamylated microtubules within the manchette to control the number of microtubules within the manchette and to disassemble the manchette at the end of spermiogenesis.

### The spastin testis interactome supports a role in the regulation of cytoskeletal architecture and key protein/vesicle transport mechanisms

To explore the mechanism of action of spastin during mammalian spermatogenesis, we identified spastin testis interaction partners using a co-immunoprecipitation (IP) mass spectrometry (MS) approach. A total of 77 proteins were significantly enriched in the spastin testis immunoprecipitates compared with controls (Table S1, Fig. S4A), including spastin itself, thus validating the IP specificity. PANTHER protein class and gene ontology analysis was conducted (Fig. S4B,C and summarised in Table S2) and functional descriptions of selected proteins of interest are summarised in Table S3. Regarding testis-specific spastin functions, a number of cytoskeletal and chromatin-related proteins were identified, which warrant further exploration in future studies (Table S3). This included regulators of the microtubule and actin cytoskeletons (e.g. DCTN2, NUDC and ARPC4) and the septin cytoskeletal filament protein, SEPT2. DCTN2 and NUDC are key regulators of the microtubule-based motor protein dynein (Table S3). Specifically, DCTN2 is a component of the dynactin complex, which is an essential co-factor for almost all functions of cytoplasmic dynein, and NUDC regulates the dynein–dynactin complex (Aumais et al., 2003). The dynein–dynactin complex has conserved roles in multiple aspects of female meiosis and mitosis, and recent work in *Drosophila* has shown that these functions extend to male meiosis (Barbosa et al., 2021). Data also suggest that the dynein–dynactin complex has roles in spermatid remodelling including at the manchette (e.g. Hayasaka et al., 2008; Lehti et al., 2017; Pleuger et al., 2020). Consistent with the phenotypes of our *Spast^KO/KO^* mice, SEPT2 and NUDC also have established functions in regulating mitotic spindle architecture and in cytokinesis. Also consistent with the phenotype observed in null germ cells, the dynein–dynactin complex facilitates vesicle transport in multiple somatic cell types (reviewed by Liu, 2017; Reck-Peterson et al., 2018), including post-Golgi transport in mouse radial glial cells (Brault et al., 2022), and SEPT2 has been shown to have roles in vesicle transport in Madin–Darby canine kidney (MDCK) cells (Spiliotis et al., 2008). Of direct relevance to the mechanism of action of spastin in severing polyglutamylated tubulin and the aberrant transport of Golgi-derived vesicles during acrosome formation herein, published data suggest that SEPT2-based filaments facilitate post-Golgi vesicle transport along polyglutamylated microtubules (Spiliotis et al., 2008), raising the possibility that SEPT2 has similar roles in male germ cells. In addition to the cytoskeletal proteins, and of potential relevance to the compromised nuclear integrity and the aberrant chromatin clumping in haploid germ cells of *Spast^KO/KO^* mice, spastin also bound multiple histone proteins that are required for chromatin remodelling including histones H1.2/H1.3 and histone H2A.Z/H2A.V (Table S3). Collectively, these data support a direct role for spastin in regulating functions and dynamics of the broader cytoskeleton, not just the microtubule component, throughout cell division and spermiogenesis.

## DISCUSSION

**Fig. 7.**
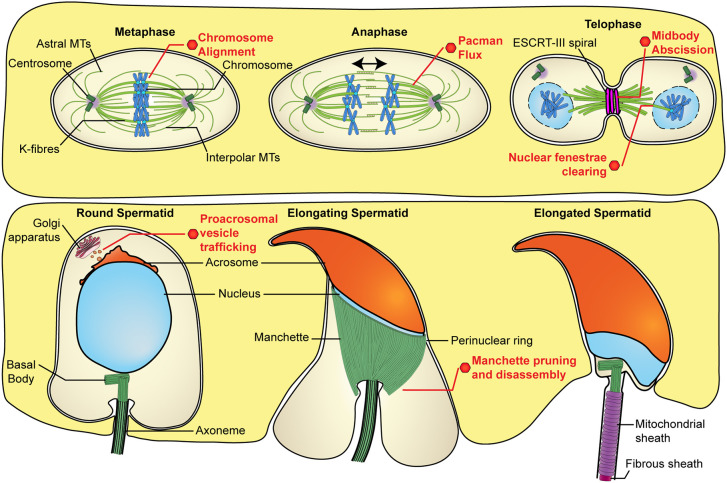
**Proposed roles of spastin during spermatogenesis.** Roles of spastin throughout the stages of spermatogenesis are visualised based on the data defined herein. Based on these data, we propose roles for spastin in chromosome alignment and segregation during meiosis, midbody abscission and nuclear envelope reformation following cell division, pro-acrosomal vesicle trafficking, and manchette pruning and disassembly. K-fibres, kinetochore fibres; MTs, microtubules.

Previously, we have shown that spermatogenesis is dependent on katanin-mediated microtubule severing (Dunleavy et al., 2021, 2017; O'Donnell et al., 2012; Smith et al., 2012). As summarised in Fig. 7, here we reveal that the microtubule-severing protein spastin is also essential for multiple aspects of male germ development, and that its loss is ultimately incompatible with sperm production (azoospermia), owing to compromised meiosis followed by a catastrophic loss of nuclear structure. Our data reveal that spastin is a regulator of meiosis, whereby it regulates metaphase and anaphase spindle function, and cytokinesis. We also reveal that spastin has essential roles in acrosome biogenesis, in ensuring spermatid nuclear integrity, and in defining the structure and function of the manchette (Fig. 7). The localisation of spastin to peri-acrosomal and manchette microtubules and the fact that the development of each of these structures is initiated in haploid germ cells (Dunleavy et al., 2019a; Pleuger et al., 2020) suggests that the phenotypic consequences of spastin loss are a primary consequence of spastin action, rather than secondary to meiotic spindle dysfunction. The possibility exists, however, that the loss of nuclear integrity in haploid male germ cells is reflective of the loss of spastin's role in the ESCRT-III-meditated sealing of the nuclear membrane during the final steps of meiotic cell division (Vietri et al., 2015).

Although no defects were apparent during *Spast^KO/KO^* male germ cell mitosis, our data establish spastin as essential for the efficient completion of male meiosis in mice. In the absence of spastin, we found increases in chromosome misalignment at the metaphase plate, in failed chromosome segregation during anaphase, and in failed or abnormal cytokinesis. Chromosome misalignment at metaphase could be due to a defect in the regulation of the length/dynamics of the microtubules that make up the bipolar spindle, or due to defects in chromosome–kinetochore attachments. In support of the latter interpretation, our interactome analysis identified that spastin interacts with NUDC, which has been shown to promote kinetochore–chromosome attachment in HeLa cells and its depletion similarly results in misaligned metaphase chromosomes (Aumais et al., 2003).

During anaphase, the failure of poleward chromosome segregation in *Spast^KO/KO^* spermatocytes suggests that spastin-mediated microtubule severing is required for the poleward shortening of spindle microtubules. This is consistent with *in vitro* data from mitosis in *Drosophila melanogaster*, wherein spastin was shown to promote poleward chromosome movement during anaphase ‘Pacman flux’, by stimulating depolymerisation of microtubule minus-ends at the spindle pole (Zhang et al., 2007). Of note, this work also identified a parallel role for the microtubule-severing enzyme Fidgetin in severing at the spindle poles during ‘Pacman flux’ (Zhang et al., 2007). It is thus possible that, in mammals, the fidgetins can compensate for spastin function during mitotic anaphase, but not in meiosis. Spastin has previously been found to localise to the spindle poles in HeLa cells, supporting a role of spastin-mediated severing in regulating other spindle types in mammals (Errico et al., 2004). It is also possible that observed failures in anaphase are related to the interaction between spastin and the dynein regulators NUDC and DCTN2. Indeed, the dynein–dynactin complex has been shown to drive chromosome segregation in *Drosophila* spermatocytes (Wu et al., 2016).

The occurrences of binucleated spermatids in the absence of spastin suggest that spastin-mediated microtubule severing is required to regulate midbody microtubule abscission during meiosis. Indeed, this is consistent with data showing that spastin severs midbody microtubules during mitosis in HeLa cells (Connell et al., 2009; Pisciottani et al., 2019; Yang et al., 2008). More commonly, however, in the absence of spastin we observed a single ‘pinched’ spermatid nucleus shared by two sister cells across an intercellular bridge. The likely explanation for this phenotype is that the ‘pinched’ nucleus occurs when there is a failure of anaphase resulting in a single, large, round spermatid nucleus, and cytokinesis then proceeds regardless of the position and size of the nucleus. A conservation of the previously elucidated role for spastin-mediated severing in establishing nuclear integrity in HeLa cells may in part explain how these nuclei become flexible enough for the pinched phenotype to occur (Vietri et al., 2015). Consistent with the results of Vietri et al., we observed an increase in DNA double-stranded breaks in *Spast^KO/KO^* mice after meiosis. Interestingly, our previous work identified frequent occurrences of binucleated spermatids when the katanin KATNB1 was lost, but never the ‘pinched’ nuclear phenotype (Dunleavy et al., 2021), indicating that spastin functions in additional aspects of anaphase and/or cytokinesis in male meiosis.

Of particular interest to the function of spastin in cytokinesis, we identified a previously unknown interaction with the septin cytoskeleton through SEPT2. Although still poorly understood, the septin cytoskeleton regulates actin and microtubule network organisation and function and has conserved roles in cytokinesis during mitosis in MDCK and HeLa cells (Spiliotis et al., 2005; Spiliotis and Nakos, 2021). Similar to the microtubule network, during cytokinesis the septin cytoskeleton is rearranged and becomes concentrated at the intercellular bridge (Russo and Krauss, 2021). The exact functions of the septin network during cytokinesis remain poorly understood, although there is growing evidence of their importance during the maturation of the intercellular bridge and in subsequent abscission of the bridge (Russo and Krauss, 2021). Another identified protein, NUDC, is also required in HeLa cells for mitosis and cytokinesis (Aumais et al., 2003).

In *Spast^KO/KO^* mice, we observed spermatid nuclei that had compromised membrane integrity. Compromised nuclear integrity first presented as deep invaginations of the nuclear membrane caused by the overlying acrosome and progressed to ruptured nuclei after manchette formation. As indicated above, we predict that compromised nuclear integrity was due to the requirement of spastin-mediated severing during nuclear membrane fission following cell division. Specifically, we hypothesise that the progressive loss of nuclear integrity seen in post-meiotic spermatids is due to the inability of the compromised nuclear membrane to withstand the pressure applied by the events of spermiogenesis, notably the nuclear-sculpting forces of the manchette (Pleuger et al., 2020).

Our data also reveal that spastin is required for the correct localisation and assembly of the acrosome during development. Without spastin, pro-acrosomal vesicles were mis-trafficked to the cytoplasm or to ectopic locations on the nuclear envelope. Although difficult to see at the light microscope level, this defect may be due to an increase in the number of microtubules emanating from the Golgi apparatus throughout the cytoplasm, and the role of spastin is supported by its localisation to the region of acrosome formation during spermiogenesis. This interpretation is supported by spastin localised to peri-acrosomal microtubules and the interaction between spastin and SEPT2. Specifically, SEPT2 facilitates post-Golgi vesicle transport along polyglutamylated microtubules (Spiliotis et al., 2008) and spastin preferentially severs polyglutamylated microtubules (Lacroix et al., 2010); therefore, we propose that spastin is required to prune SEPT2-associated polyglutamylated microtubules to target pro-acrosomal vesicle trafficking to the apical pole of the nucleus. In the absence of spastin action, we predict that the microtubule network between the Golgi and the nuclear membrane would remain unfocused and thus vesicles would become mistargeted. Such an interpretation is analogous to that made in previous work on mouse neuronal development, which found that spastin is required for the pruning of axon branches during neurogenesis (Brill et al., 2016), and that microtubule-based axonal transport is disrupted in the absence of spastin (Tarrade et al., 2006).

Our data establish spastin is an essential regulator of manchette microtubule density, length and disassembly, and manchette–nuclear attachment. Previous research found that spastin preferentially severs polyglutamylated microtubules (Lacroix et al., 2010; Valenstein and Roll-Mecak, 2016) and that loss of spastin leads to an increase in polyglutamylated tubulin (Magiera et al., 2018). Our results support a role for spastin in regulating the accumulation and length of manchette microtubules as we found an increase in polyglutamylated microtubules within *Spast^KO/KO^* manchettes. Our previous work identified that the katanin KATNAL2 and its regulatory subunit, KATNB1, are also important for manchette movement and length, indicating that a suite of microtubule-severing enzymes are required to regulate different aspects of the manchette (Dunleavy et al., 2021; 2017).

To our knowledge, this is the first time that many *in vitro* phenotypes resulting from a loss of spastin have been confirmed in an *in vivo* model. We have shown a phenotype consistent with work showing that spastin is required for the completion of nuclear envelope re-formation and for midbody abscission and have highlighted the biological relevance of these roles during spermatogenesis. Additionally, aspects of the manchette phenotype observed are unique and suggest a distinct role for spastin in the regulation of complex microtubule-based structures. This work establishes spastin as a key regulator of microtubule dynamics during spermatogenesis, and many microtubule-dependent processes are disrupted without its action. Our work provides a better understanding of the disrupted cell dynamics observed in cases of hereditary spastic paraplegia, increases our understanding of the role of microtubule regulation in spermatogenesis, and may ultimately inform fertility care for individuals carrying *SPAST* loss-of-function genetic variants.

## MATERIALS AND METHODS

### Animal ethics statement

All animal procedures were performed with the approval of the Monash University Animal Experimentation Ethics Committee (Project 20019) or the University of Melbourne Ethics Committee (Project 20640) and were consistent with the requirements set out in the Australian National Health and Medical Research Council (NHMRC) Guidelines on Ethics in Animal Experimentation.

### Mouse model production and phenotypic analysis

The mouse model used for this study was first described by Brill et al. (2016) who showed that spastin was involved in dendritic pruning. In brief, the *Spast^tm1a(KOMP)Wtsi^* targeting vector (PG00198_Z_2_G10) was generated by the trans-NIH Knockout Mouse Project (www.komp.org). The construct contains a splice site acceptor and a polyadenylation sequence resulting in truncation following exon 4 (ENSMUSE00000137944) of *Spast* (ENSMUSG00000024068). Mice were maintained on a C57BL/6 background. Wild-type littermates (*Spast^WT/WT^*) were used as controls for *Spast* knockout mice (*Spast^KO/KO^*), and all male mice used for analysis were adult (≥10 weeks of age). Mouse genotypes were identified from tail biopsies using real-time PCR with probes designed for each allele (Transnetyx, Cordova, TN, USA). *Spast* mRNA levels in *Spast^KO/KO^* mice were tested using qPCR on whole testis tissue as described below.

### Quantitative qPCR

Whole testes were homogenised, and total RNA was extracted using TRIzol reagent (Life Technologies) and cDNA synthesised using SuperScript III reverse transcriptase (Life Technologies). To verify the truncation of the *Spast* gene in the *Spast^KO/KO^* mouse line, PCR primer sets were designed that spanned part of exon 4 and exon 5 (forward 5′-TAACCTGACATGCCGCAATG-3′ and reverse 5′-ACAAACCACTGCAACTAGGC-3′). qPCR was performed using SYBR Select Master Mix (Applied Biosystems). Each reaction was performed in triplicate, and on three biological replicates per genotype, on an Applied Biosystems QuantStudio 3 real-time PCR system. DNA was denatured at 95°C for 2 min, followed by 35 cycles of 95°C for 30 s and then 60°C for 1 min. *Ppia* was amplified simultaneously as an internal control (forward primer 5′-CGTCTCCTTCGAGCTGTTT-3′ and reverse primer 5′-CCCTGGCACATGAATCCT-3′). All results were normalised to the expression of *Ppia.* Differential expression was analysed using the 2ΔΔ^CT^ method (Livak and Schmittgen, 2001).

### Fertility characterisation

The fertility of the *Spast^KO/KO^* mouse line was characterised as described by Houston et al. (2021). Fertility tests used male mice ≥10 weeks of age, in which males were mated with two wild-type females (≥6 weeks of age). Females were monitored for copulatory plugs as an indication of successful mating and the number of pups born per copulatory plug was recorded. Testis daily sperm production (DSP) and total epididymal sperm content (*n*≥3 mice/genotype) were determined using the Triton X-100 nuclear solubilisation method described by Dunleavy et al. (2021). Briefly, homogenisation of whole testes or epididymides in DSP buffer (0.9% NaCl, 0.01% NaN_3_, 0.05% Triton X-100; 1 ml per testis or epididymis) was performed to isolate condensed, elongated spermatids. To remove sperm tails and any remaining uncondensed cells, samples were sonicated using a 125W probe sonicator (Q125 Sonicator, Qsonica) set at 30% amplitude for 3×10 s cycles with 10 s intervals between cycles. The number of elongated spermatids per testis and epididymis was determined using a haemocytometer. Testis DSP was calculated by multiplying the number of sperm per testis by 4.84 (the number of days elongated spermatids reside in the mouse testis; Russell, 1990).

Testes and epididymides were fixed in Bouin's fixative and processed into paraffin for histological examination. PAS and Haematoxylin staining was used to visualise the male reproductive tract histology (*n*≥3 mice/genotype). Germ cell apoptosis was evaluated by immunostaining for cleaved caspases 3 and 9 and counterstaining for Haematoxylin as previously described (O'Bryan et al., 2013). The number of caspase-positive cells in a minimum of 100 randomly selected seminiferous tubules per mouse was quantified and statistical analysis was performed as detailed below (*n*=3 mice/genotype). Metaphase chromosome alignment defects during meiosis and acrosome abnormalities were quantified using PAS-stained testis sections. All metaphase spermatocytes in five stage XII seminiferous tubules per mouse were scored as having either normal, dispersed or misaligned chromosome morphology. All cap-phase spermatids in five seminiferous tubules per mouse were scored as having normal or abnormal acrosome morphology. To quantify spermatid nuclear integrity defects, a minimum of 48 elongating spermatids per mouse were scored as either intact or abnormal using transmission electron micrographs prepared as detailed below. To quantify manchette abnormalities, testis sections were immunolabelled for α-tubulin as detailed below, and all step 12 spermatids in five stage XII seminiferous tubules per mouse were scored as normal or abnormal. Statistical analysis of meiotic chromosome alignment, acrosome morphology, nuclear integrity and manchette morphology defects was conducted as detailed below (*n*=3 mice/genotype).

### Transmission electron microscopy

To analyse the ultrastructure of the seminiferous tubules, partially decapsulated testes were processed for transmission electron microscopy as described by Dunleavy et al. (2021). Ultrathin sections were cut on a Reichert Jung Ultracut Microtome and placed on 100×100 square copper grids (ProSciTech). Sections were analysed using a Jeol JEM-1400 Plus transmission electron microscope at the Monash University Ramaciotti Centre for Cryo-Electron Microscopy (Monash University, Clayton, Australia).

### Antibodies

Primary antibodies used included those against α-tubulin (T5168, Sigma-Aldrich, ascites fluid, 1:5000; ab4074, Abcam, 1 μg ml^−1^), β-tubulin (ab21057, Abcam, 1 µg ml^−1^), spastin (ab31850, Abcam, 10 µg ml^−1^; Fig. S1A-C), polyglutamylated tubulin B3 (T9822, Sigma-Aldrich, 2 µg ml^−1^), γH2AX (05-636, Millipore, 0.1 µg ml^−1^), cleaved caspase 3 (9664, Cell Signaling Technology, 0.5 μg ml^−1^) and cleaved-caspase 9 (9509, Cell Signaling Technology, 1 μg ml^−1^). Secondary antibodies included Alexa Fluor 488 donkey anti-goat (A11055, Invitrogen), Alexa Fluor 555 donkey anti-goat (A21432, Invitrogen), Alexa Fluor 555 donkey anti-mouse (A31570, Invitrogen), Alexa Fluor 647 donkey anti-mouse (A31571, Invitrogen) and Alexa Fluor 647 donkey anti-rabbit (A31573, Invitrogen). Parallel sections were processed in the absence of a primary antibody to control for secondary antibody specificity.

### Germ cell isolation

STAPUT-purified germ cells were prepared as detailed by Dunleavy et al. (2019b). For immunofluorescence labelling, cells were fixed in 4% paraformaldehyde for 20 min.

### Immunochemistry

Five-micrometre-thick sections were cut from paraffin blocks and dewaxed prior to antigen retrieval by microwaving the sections in 10 mM citrate buffer (pH 6.0) for 16 min as previously described (Jamsai et al., 2008). For colorimetric immunohistochemistry, endogenous peroxidase activity was blocked with 3% H_2_O_2_ in H_2_O for 5 min, and non-specific antibody binding was minimised by blocking with CAS block (Invitrogen) for at least 30 min. Primary antibodies were diluted in Dako antibody diluent (S0809, Dako) and incubated overnight at 4°C. Dako EnVision Polymer Dual link system-HRP (K4063, Dako) was applied undiluted for 1 h at room temperature. Dako liquid DAB+ substrate chromogen (K3468, Dako) was applied to samples for 1 min followed by immediate submersion in water. Sections were counterstained with Haematoxylin then dehydrated and mounted with DPX (44581, Sigma-Aldrich).

For immunofluorescence labelling, testis sections were dewaxed and antigen retrieval conducted as above. For both testis sections and purified germ cells, non-specific antibody binding was minimised by incubating sections in CAS Block (Invitrogen). Primary antibodies were diluted in Dako antibody diluent (S0809, Dako) and incubated on sections overnight at 4°C. Secondary antibodies were diluted 1:500 in PBS and incubated on sections at room temperature for 1 h. DNA was visualised using 1 µg ml^−1^ 4′,6-diamidino-2-phenylindole (DAPI, Invitrogen). Acrosomes were visualised using 0.5 µg ml^−1^ lectin peanut agglutinin (PNA) Alexa Fluor 488 conjugate (L21409, Life Technologies). Sections were mounted under Dako fluorescence mounting medium and glass coverslips (GM304, Dako).

Immunofluorescent images were taken with a Leica TCS SP8 confocal microscope (Leica Microsystems) at the University of Melbourne Biological Optical Microscopy Platform. All images were taken using a 63×/1.40 HC PL APO CS2 oil immersion objective. *z*-stacks of testis sections were collected at 0.3 µm intervals and assembled into maximum intensity projections in ImageJ and processed using ImageJ v2.1.0.

### Proteomic analysis to identify the spastin testis interactome

For IP assays, wild-type mouse testes (*n*=3 per IP assay) were lysed in 1% NP-40 (in PBS) supplemented with protease inhibitor cocktail (Calbiochem, 539134). Co-IP of SPAST and its binding partners was performed using the Pierce Co-immunoprecipitation Kit (Thermo Scientific, 26149) as per the manufacturer's instructions, in combination with the SPAST antibody (Abcam, ab31850, 5 μg per IP column). To minimise non-specific binding, testis protein lysates were pre-cleared by incubation with 400 μl Pierce control agarose resin slurry for 4 h at 4°C with gentle rocking. For each pre-cleared testis lysate, 4 mg was then loaded into two IP columns, one which contained the target antibody covalently coupled to the amine reactive Pierce AminoLink Plus Coupling Resin and one which contained the target antibody and a non-amine reactive Pierce Control Agarose Resin (negative control), and incubated overnight at 4°C. Lysate flow-through was removed, columns were washed, and bound protein eluted and collected as per the manufacturer's instructions. The eluted proteins were neutralised with 1.5 M Tris-HCl (pH 8.8), reduced with tris (2-carboxyethyl) phosphine (TCEP), then digested by incubation with trypsin overnight at 37°C. Peptide samples were freeze-dried and resuspended in 2% acetonitrile (ACN)/0.1% formic acid (FA) (diluted in MilliQ water) before concentrated FA was added to adjust the pH to <3. Samples were purified using reverse-phase chromatography C18 stage tips, which aim to desalt and fractionate in-solution peptides at an acidic pH. The tips were initially activated with 50% ACN/0.1% FA and equilibrated with 0.1% FA, before samples were loaded into them and washed with 0.1% FA. Peptides were eluted with 50% ACN/0.1% FA, freeze-dried, and resuspended in 2% ACN/0.1% FA.

The purified peptide samples were analysed by nano liquid chromatography coupled to tandem mass spectrometry (LC-MS/MS) at the University of Melbourne Mass Spectrometry and Proteomics Facility, using an Orbitrap Eclipse Tribrid Mass Spectrometer (Thermo Fisher Scientific) equipped with a nano ESI interface coupled to an Ultimate 3000 nano HPLC (Thermo Fisher Scientific). Peptides were separated using an Acclaim PepMap RSLC analytical column (C18, 100 Å, 75 μm×50 cm, Thermo Fisher Scientific, USA) and Acclaim PepMap trap column (75 μm×2 cm, C18, 100 Å). The enrichment column was injected with the tryptic peptides (3 µl) at an isocratic flow of 5 μl/min of 2% v/v CH_3_CN containing 0.05% v/v aqueous trifluoroacetic acid for 6 min, applied before the enrichment column was switched in-line with the analytical column. The eluents were 0.1% v/v aqueous formic acid and 5% v/v dimethyl sulfoxide (DMSO) (solvent A) and 0.1% v/v formic acid and 5% DMSO in acetonitrile (solvent B). The gradient was at 300 nl min^−1^ from (1) 0-6 min, 3% solvent B; (2) 6-35 min, 3-23% solvent B; (3) 35-45 min, 23-40% solvent B; (4) 45-50 min, 40-80% solvent B; (5) 50-55 min, 80-80% solvent B; (6) 55-55.1 min, 80-3% solvent B; (7) 55.1-65 min, 3-3% solvent B. The column oven was maintained at 50°C throughout the analysis. The Eclipse Orbitrap mass spectrometer was operated in the data-dependent mode, wherein full MS^1^ spectra were acquired in a positive mode over the range of m/z 375-1500, with spray voltage at 1.9 kV, source temperature at 275°C, MS^1^ at 120,000 resolution and normalised AGC target of 100% and maximum ion injection time of 50 ms. The top 3 s method was used and selecting peptide ions with charge states of ≥2-7 and intensity thresholds of ≥5E4 were isolated for MS/MS. The isolation window was set at 1.6 m/z, and precursors were fragmented using higher energy C-trap dissociation (HCD) at a normalised collision energy of 30%, a resolution of 15,000, a normalised AGC target of 100% and automated IT time.

The raw MS data were analysed with the MaxQuant software suite for the identification and quantification of peptides/proteins from the Mouse SwissProt database. Trypsin/P was set as the protease with a maximum of two missed cleavages. Statistical analysis was conducted with Perseus software considering protein and peptide-to-spectrum match (PSM) false discovery rates (FDR) both set at <0.01. The majority UniProt ID(s) for each significantly enriched protein group were then analysed using PANTHER gene ontology and protein classification (Thomas et al., 2022). The mass spectrometry proteomics data have been deposited to the ProteomeXchange Consortium via the PRIDE (Perez-Riverol et al., 2022) partner repository with the dataset identifier PXD038779.

### Statistics and reproducibility

Statistical analysis of the germ cell apoptosis data was performed in R version 3.5.1. Generalised linear mixed models (GLMs) were used to compare the number of caspase-positive cells per tubule between genotypes. For each model, Akaike information criterion (AIC) estimates were used to select the most appropriate error distribution and link functions (i.e. Poisson, negative binomial, zero-inflated Poisson, zero-inflated negative binomial) using the glmer function (lme4 package; Bates et al., 2015) and the glmmTMB function (glmmTMB package; Brooks et al., 2017). For all models, a zero-inflated negative binomial distribution (fitted with glmmTMB, using the ziformula argument) was selected as the most appropriate error distribution and link function (i.e. had the lowest AIC score).

All other statistical analysis was performed in GraphPad Prism version 9.3. The statistical significance of differences between two groups was determined using an unpaired Student's *t*-test, with significance defined as *P*<0.05. For each group a minimum of *n*=3 individuals per group were analysed.

## Supplementary Material

10.1242/develop.201183_sup1 Supplementary information
