# Supplementary material for: Spastin is an essential regulator of male meiosis, acrosome formation, manchette structure and nuclear integrity
Source: Development. 2023 Mar 30;150(6):dev201183. doi: 10.1242/dev.201183 (PMC10112905; doi:10.1242/dev.201183)
Supplement: Supplementary information [file develop-150-201183-s1.pdf]

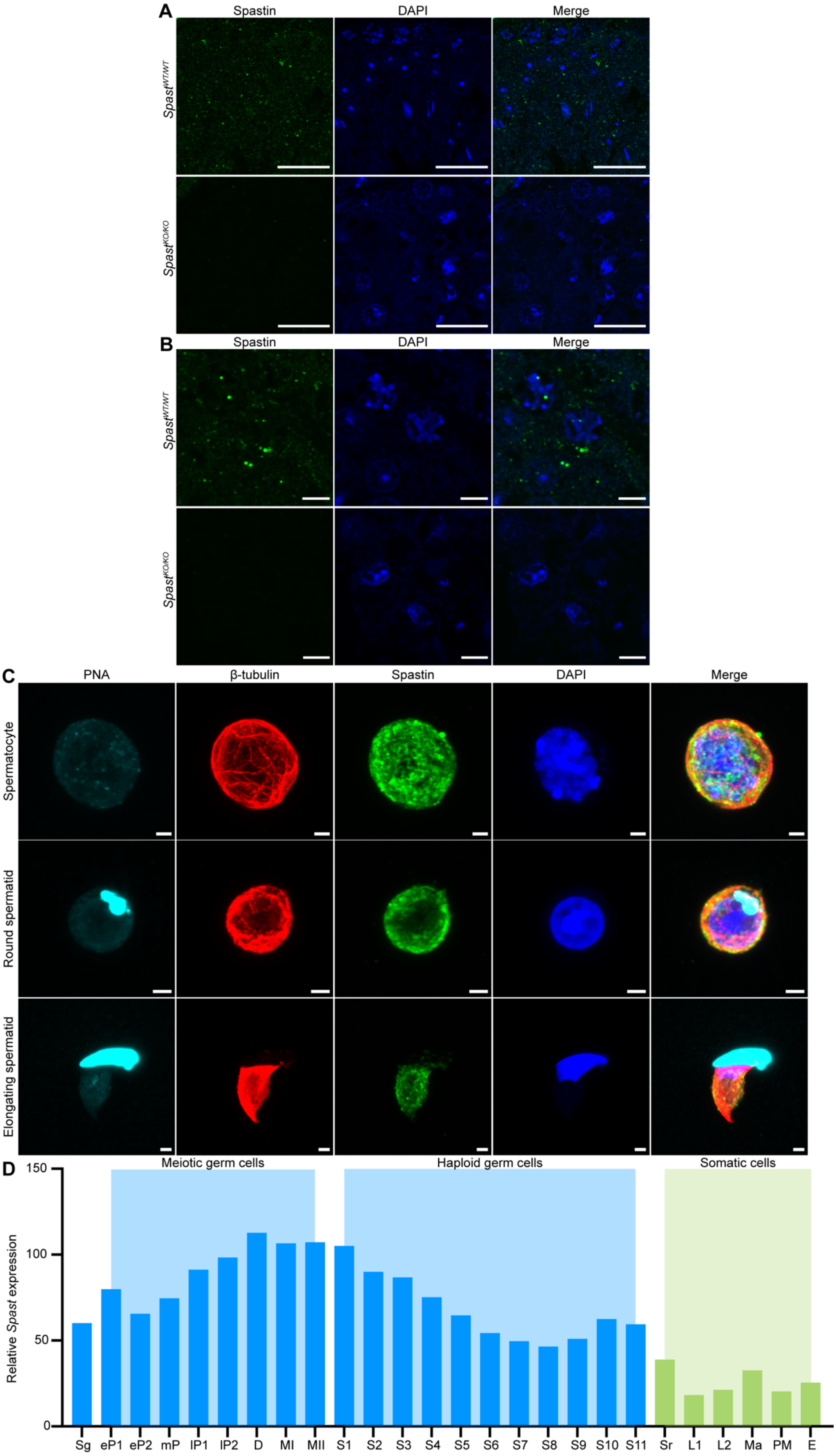

Development • Supplementary information

Fig. S1. Spastin is ubiquitously expressed throughout spermatogenesis. Lower (**A**) and higher magnification (**B**) *Spast*$^{WT/WT}$ and *Spast*$^{KO/KO}$ testis sections immunolabeled for spastin (green). Nuclei are counterstained with DAPI (blue). Scale bars in **A** = 20 µm **B** = 5 µm (**B**) Analysis of spastin (green) localisation in purified wildtype male germ cells (**C**) reveals spastin is present throughout the cytoplasm of spermatocytes and spermatids wherein it colocalises with microtubules (marked by β-tubulin (red)). The acrosome is visualised using PNA (cyan) and the nucleus with DAPI (blue). Scale bars of **C** = 2 µm. (**D**) Testicular single cell RNA sequencing data for the mouse show *Spast* expression across major cell types in the adult mouse testis present throughout all stages of spermatogenesis, peaking during meiotic divisions. Data were generated by (Ernst et al., 2019). In **D** X-axis left to right: Sg = spermatogonia, eP1-eP2 = early pachytene 1-2 spermatocytes, D = diplotene spermatocytes, MI = meiosis I, MII = meiosis II, S1-S11 = step 1-11 spermatids, Sr = Sertoli cell, L1-2 = Leydig cells 1-2, Ma = macrophages, PM = peritubular myoid cells, E = endothelial cells.

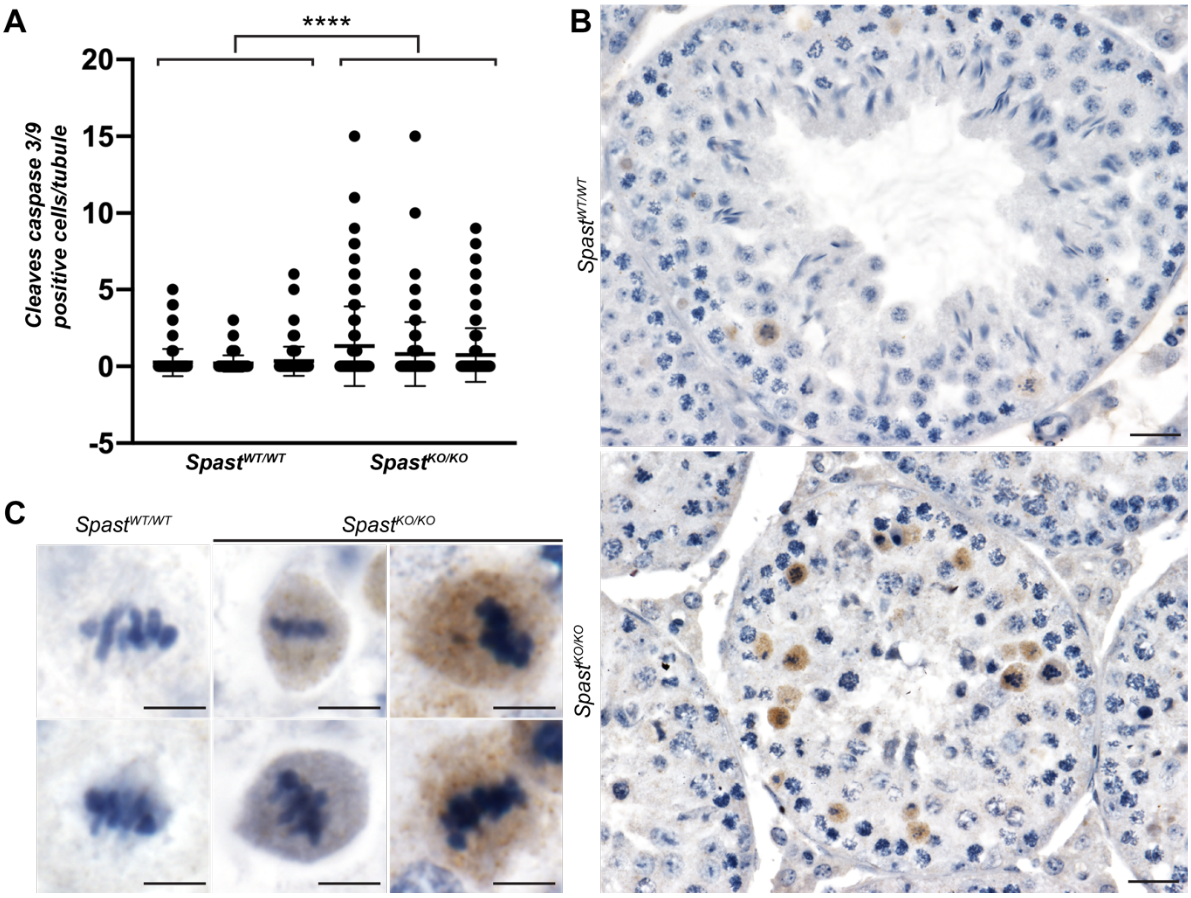

Fig. S2. Loss of spastin results in an increase of germ cell apoptosis. Apoptosis of germ cells was assessed using immunohistochemical staining of cleaved-caspase 3 and 9. The average number of cleaved-caspase 3 and/or 9 positive cells per seminiferous tubule for is graphed in (**A**). Each column represents a single mouse and lines represent mean±s.d. A minimum of 100 randomly selected seminiferous tubules per mouse were counted. A statistically significant increase in germ cell apoptosis was found in $Spast^{KO/KO}$ mice compared to $Spast^{WT/WT}$mice, **** $p<0.0001$. Representative seminiferous tubules and cells for each genotype can be seen in (**B-C**). Scale bars in **B** = 20µm, scale bars in **C** = 5µm.

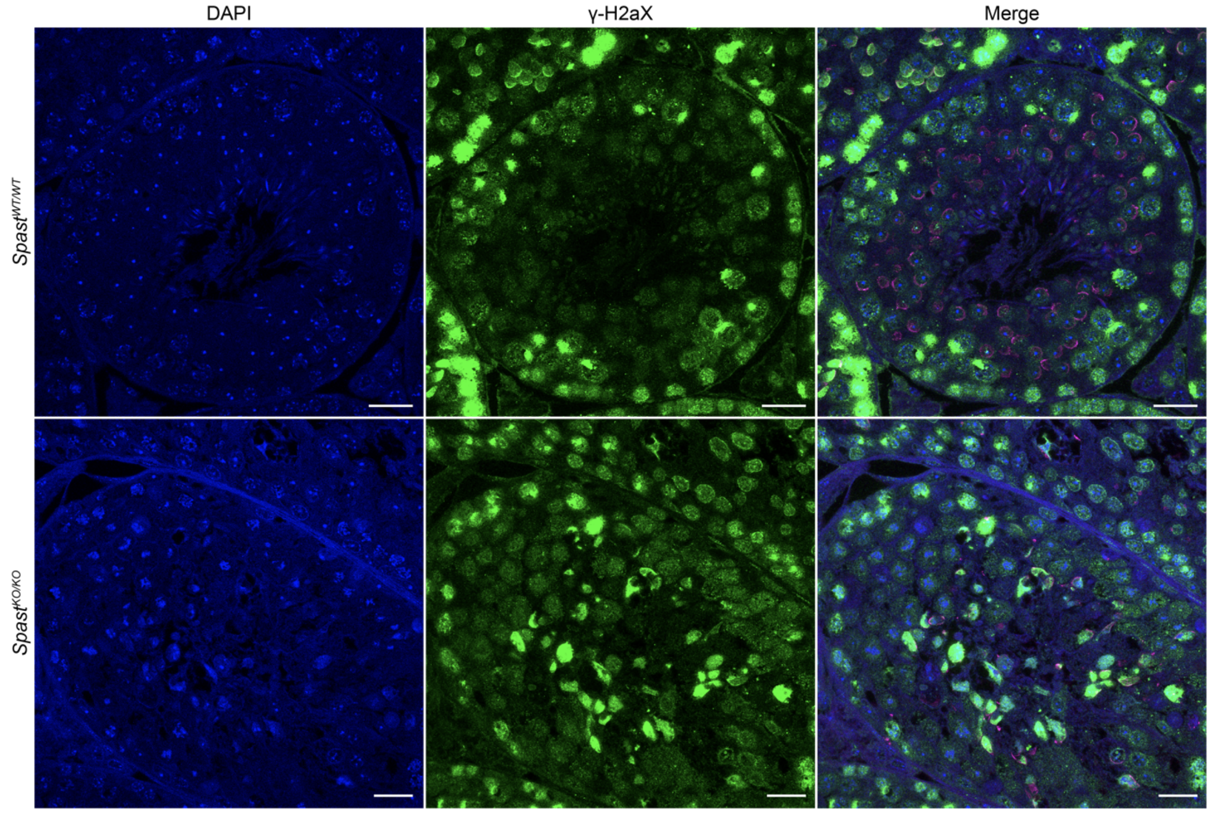

Fig. S3. The loss of spastin results in an increase in DNA double stranded breaks in post-meiotic germ cells. Staining for γ-H2aX (green) to identify double stranded breaks in DNA in *Spast*$^{WT/WT}$ and *Spast*$^{KO/KO}$ testis sections. Nuclei were counterstained with DAPI (blue) and acrosomes with PNA (magenta). Positive γ-H2aX staining was observed in post-meiotic germ cells (spermatids) from *Spast*$^{KO/KO}$ but not *Spast*$^{WT/WT}$ mice. Post-meiotic germ cells may be identified by their location closer to the tubule lumen and by the presence of an acrosome, shown as magenta in the merged image. Scale bar = 20µm.

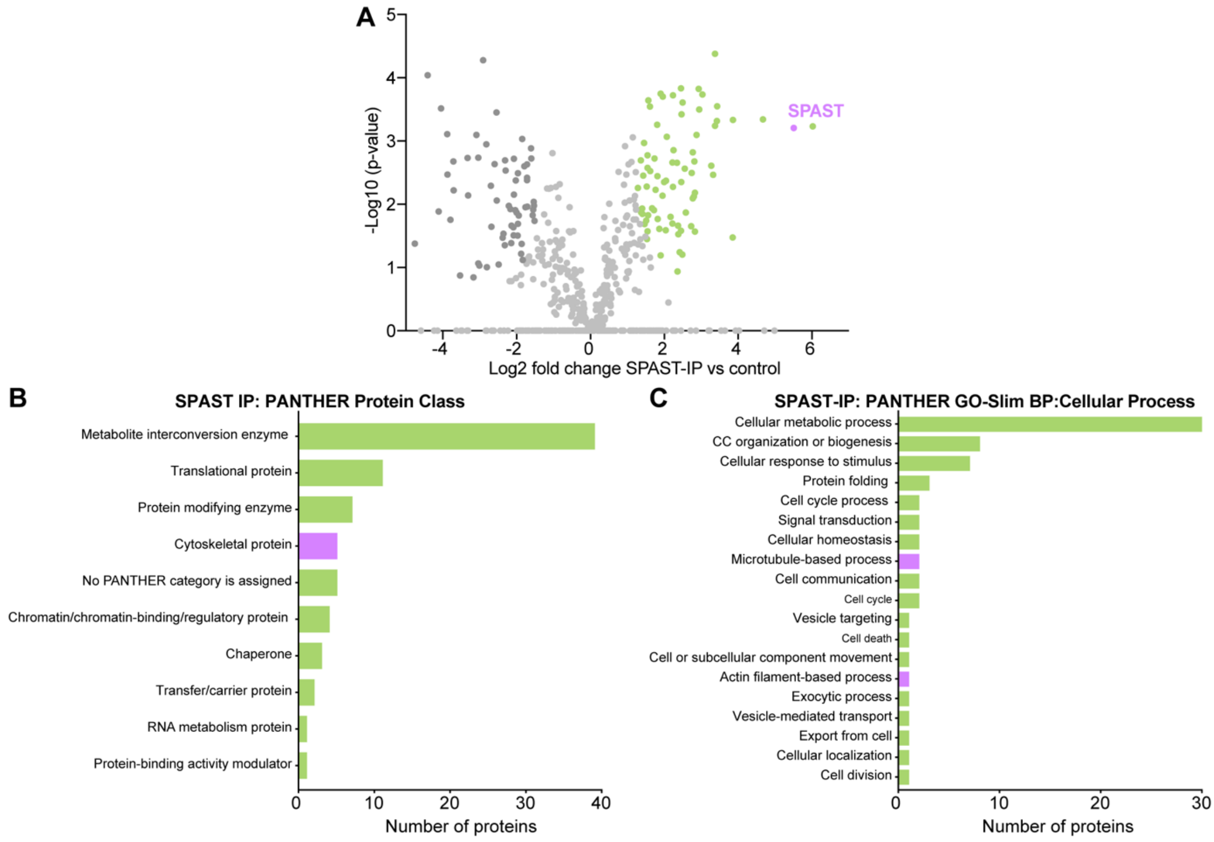

Fig. S4. Identification of spastin testis interaction partners. (**A**) Volcano blot showing the statistical enrichment of protein groups identified in the spastin IP-MS assay. All protein groups identified, IP-MS measurements and statistical analysis is provided in Table S1. Protein groups shown in green or pink, represent those significantly enriched in the spastin IP. For the IP-MS assay n=3 biological replicates were assessed. (**B-C**) PANTHER Protein Class and Gene Ontology analysis was used to analyse the proteins significantly enriched in spastin IPs. The PANTHER Protein Class and the of the PANTHER GO-Slim Biological Process (BP) Cellular Process (GO:0009987) sub-classification are summarised in **B** and **C** respectively. PANTHER was not able to assign all proteins identified. Detailed PANTHER Protein Class and GO-Slim analysis is provided in Table S2. CC = Cellular component.

Table S1. Significantly enriched proteins identified in spastin testis co-IP experiments with subsequent MS analysis. Relates to Fig S4 and Tables S2 and S3.

Click here to download Table S1

Table S2. PANTHER analysis of proteins identified by MS as significantly enriched in the spastin testis co-immunoprecipitates. Relates to Fig S4 and Tables S1 and S3.

Click here to download Table S2

Development • Supplementary information

Table S3. Functional descriptions of selected spastin candidate testis binding proteins of relevance to the observed *SpastKO/KO* mouse phenotype.

Gene name(s)	Protein name(s)	Function
**Cytoskeletal-related proteins**		
Arpc4	Actin-related protein 2/3 complex subunit 4	Component of the ARP2/3 complex, which regulates (F-)actin filament polymerisation and mediates the formation of branched F-actin networks. Components are enriched in ectoplasmic specialisations (junctions between Sertoli cells and male germ cells), and data supports roles in blood-testis barrier integrity, spermiogenesis and spermiation (Liu et al., 2018, Lie et al., 2010, Li et al., 2015, O'Donnell et al., 2011).
Sept2	Septin-2	Filament forming cytoskeletal element that is required for normal organisation of the actin cytoskeleton (Kremer et al., 2007), and for mitosis (Spiliotis et al., 2005). Forms a complex at the sperm annulus which is required for the structural integrity of the sperm tail during post meiotic differentiation (Kuo et al., 2015). Required to maintain polyglutamylated microtubules required for cell polarity and vesicular transport in epithelial cells (Spiliotis et al., 2008)
Nudc	Nuclear migration protein nudC	Nuclear movement protein that associates with dynein-dynactin complexes, tubulin and microtubule organising centres. Established roles in human mitosis including spindle formation, chromosome segregation and cytokinesis (Zhou et al., 2003, Chen et al., 2015). Role in cell proliferation in humans (Aumais et al., 2003). NUDC has not

Development • Supplementary information

		previously been investigated in the context of male fertility.
Dctn2	Dynactin subunit 2	Essential components of the dynactin complex. Dynactin binds dynein and microtubules, to aid dynein-mediated cargo transport along microtubules. Dynactin drives chromosomes segregation in *C. elegans* spermatocytes (Barbosa et al., 2020) and its disruption leads to male infertility in *Drosophila* (Wu et al., 2016).
**Chromatin-binding or chromatin-regulatory proteins**		
H1-2/ H1-3	Histone H1.2/ Histone H1.3	Binds to linker DNA between nucleosomes to enable the formation of higher-order structures (Fan et al., 2003). Acts as a regulator of individual gene transcription through chromatin remodelling (Fan et al., 2005).
H2az1/H2az2	Histone H2A.Z/ Histone H2A.V	Variant of histone H2A present in a subset of nucleosomes. As a histone plays essential roles in transcription regulation, DNA repair, DNA replication and chromosomal stability. May be required for chromosome segregation during cell division (Rangasamy et al., 2004). Required for early mammalian development (Faast et al., 2001).

Development • Supplementary information

Supplementary references

Aumais, J. P., Williams, S. N., Luo, W., Nishino, M., Caldwell, K. A., Caldwell, G. A., Lin, S. H. and Yu-Lee, L. Y. (2003). Role for NudC, a dynein-associated nuclear movement protein, in mitosis and cytokinesis. *J Cell Sci* **116**, 1991-2003.

Barbosa, D. J., Teixeira, V., Duro, J., Carvalho, A. X. and Gassmann, R. (2020). Dyneindynactin segregate meiotic chromosomes in C. elegans spermatocytes. *Development* **148**.

Chen, D., Ito, S., Yuan, H., Hyodo, T., Kadomatsu, K., Hamaguchi, M. and Senga, T. (2015). EML4 promotes the loading of NUDC to the spindle for mitotic progression. *Cell Cycle* **14**, 1529-1539.

Ernst, C., Eling, N., Martinez-Jimenez, C. P., Marioni, J. C. and Odom, D. T. (2019). Staged developmental mapping and X chromosome transcriptional dynamics during mouse spermatogenesis. Nature Communications 10, 1251.

Faast, R., Thonglairoam, V., Schulz, T. C., Beall, J., Wells, J. R. E., Taylor, H., Matthaei, K., Rathjen, P. D., Tremethick, D. J. and Lyons, I. (2001). Histone variant H2A.Z is required for early mammalian development. *Current Biology* **11**, 1183-1187.

Fan, Y., Nikitina, T., Morin-Kensicki, E. M., Zhao, J., Magnuson, T. R., Woodcock, C. L. and Skoultchi, A. I. (2003). H1 linker histones are essential for mouse development and affect nucleosome spacing in vivo. *Mol Cell Biol* **23**, 4559-4572.

Fan, Y., Nikitina, T., Zhao, J., Fleury, T. J., Bhattacharyya, R., Bouhassira, E. E., Stein, A., Woodcock, C. L. and Skoultchi, A. I. (2005). Histone H1 depletion in mammals alters global chromatin structure but causes specific changes in gene regulation. *Cell* **123**, 1199-1212.

Kremer, B. E., Adang, L. A. and Macara, I. G. (2007). Septins regulate actin organization and cell-cycle arrest through nuclear accumulation of NCK mediated by SOCS7. *Cell* **130**, 837-850.

Kuo, Y. C., Shen, Y. R., Chen, H. I., Lin, Y. H., Wang, Y. Y., Chen, Y. R., Wang, C. Y. and Kuo, P. L. (2015). SEPT12 orchestrates the formation of mammalian sperm annulus by organizing core octameric complexes with other SEPT proteins. *J Cell Sci* **128**, 923-934.

Development • Supplementary information

Li, N., Mruk, D. D., Tang, E. I., Wong, C. K. C., Lee, W. M., Silvestrini, B. and Cheng, C. Y. (2015). Formins: Actin nucleators that regulate cytoskeletal dynamics during spermatogenesis. *Spermatogenesis* **5**.

Lie, P. P. Y., Chan, A. Y. N., Mruk, D. D., Lee, W. M. and Cheng, C. Y. (2010). Restricted Arp3 expression in the testis prevents blood-testis barrier disruption during junction restructuring at spermatogenesis. Proceedings of the National Academy of Sciences of the United States of America **107**, 11411-11416.

Liu, L. Q., Zhang, Y. B., Chang, X. L., Li, R., Wu, C. H., Tang, L. M. and Zhou, Z. J. (2018). Fluorochloridone perturbs blood-testis barrier/Sertoli cell barrier function through Arp3-mediated F-actin disruption. *Toxicology Letters* **295**, 277-287.

O'Donnell, L., Nicholls, P. K., O'Bryan, M. K., McLachlan, R. I. and Stanton, P. G. (2011). Spermiation: The process of sperm release. *Spermatogenesis* **1**, 14-35.

Rangasamy, D., Greaves, I. and Tremethick, D. J. (2004). RNA interference demonstrates a novel role for H2A.Z in chromosome segregation. *Nat Struct Mol Biol* **11**, 650-655.

Spiliotis, E. T., Hunt, S. J., Hu, Q., Kinoshita, M. and Nelson, W. J. (2008). Epithelial polarity requires septin coupling of vesicle transport to polyglutamylated microtubules. *J Cell Biol* **180**, 295-303.

Spiliotis, E. T., Kinoshita, M. and Nelson, W. J. (2005). A mitotic septin scaffold required for Mammalian chromosome congression and segregation. *Science* **307**, 1781-1785.

Wu, C. H., Zong, Q., Du, A. L., Zhang, W., Yao, H. C., Yu, X. Q. and Wang, Y. F. (2016). Knockdown of Dynamitin in testes significantly decreased male fertility in Drosophila melanogaster. *Developmental Biology* **420**, 79-89.

Zhou, T., Aumais, J. P., Liu, X., Yu-Lee, L. Y. and Erikson, R. L. (2003). A role for Plk1 phosphorylation of NudC in cytokinesis. *Dev Cell* **5**, 127-138.
