# Supplementary material for: Spastin is an essential regulator of male meiosis, acrosome formation, manchette structure and nuclear integrity
Source: Development. 2023 Mar 30;150(6):dev201183. doi: 10.1242/dev.201183 (PMC10112905; doi:10.1242/dev.201183)
Supplement: Supplementary file 1 [file develop-150-201183_review_history.pdf]

Spastin is an essential regulator of male meiosis, acrosome formation, manchette structure and nuclear integrity

Samuel R. Cheers, Anne E. O'Connor, Travis K. Johnson, D. Jo Merriner, Moira K. O'Bryan and Jessica E. M. Dunleavy
DOI: 10.1242/dev.201183

Editor: Haruhiko Koseki

Review timeline

Original submission:	16 September 2022
Editorial decision:	4 November 2022
First revision received:	9 January 2023
Editorial decision:	6 February 2023
Second revision received:	20 February 2023
Accepted:	24 February 2023

Original submission

First decision letter

MS ID#: DEVELOP/2022/201183

MS TITLE: Spastin is an essential regulator of male meiosis, acrosome formation, manchette structure and nuclear integrity

AUTHORS: Samuel R Cheers, Anne E O'Connor, Travis K Johnson, D. Jo Merriner, Moira K O'Bryan, and Jessica EM Dunleavy

I have now received all the referees' reports on the above manuscript, and have reached a decision. The referees' comments are appended below, or you can access them online: please go to BenchPress and click on the 'Manuscripts with Decisions' queue in the Author Area.

As you will see, the referees express considerable interest in your work, but have some significant criticisms and recommend a substantial revision of your manuscript before we can consider publication. If you are able to revise the manuscript along the lines suggested, which may involve further experiments, I will be happy receive a revised version of the manuscript. It is particularly important to show subcellular distribution of Spastin as requested by the Reviewer 1. Your revised paper will be re-reviewed by one or more of the original referees, and acceptance of your manuscript will depend on your addressing satisfactorily the reviewers' major concerns. Please also note that Development will normally permit only one round of major revision. If it would be helpful, you are welcome to contact us to discuss your revision in greater detail. Please send us a point-by-point response indicating your plans for addressing the referee's comments, and we will look over this and provide further guidance.

Please attend to all of the reviewers' comments and ensure that you clearly highlight all changes made in the revised manuscript. Please avoid using 'Tracked changes' in Word files as these are lost in PDF conversion. I should be grateful if you would also provide a point-by-point response detailing how you have dealt with the points raised by the reviewers in the 'Response to Reviewers' box. If you do not agree with any of their criticisms or suggestions please explain clearly why this is so.

© 2023. Published by The Company of Biologists under the terms of the Creative Commons Attribution License (https://creativecommons.org/licenses/by/4.0/).

Reviewer 1

Advance summary and potential significance to field

The paper by Cheers et al entitled “Spastin is an essential regulator of male meiosis, 1 acrosome formation manchette structure and nuclear integrity” describes the establishment and analysis of a Spastin-deficient mouse line. The authors report many pathophysiological changes and claim several to be intrinsically primary defects of loss of Spastin.
While it is an interesting phenotype (which has been described in brief before by Tarrade et al. 2006) the authors need to address some points before the ms should be considered for publication.

Comments for the author

One major point is the lack of an appropriate antibody staining, which would clearly demonstrate the localization of Spastin during spermiogenesis. If Spastin can be detected in acrosome, manchette and nuclear membranes, that the argument, that the defects observed in ko animals are primary defects might hold. Without this, one might argue, that loss of Spastin leads to meiosis defects and, as a consequence to initiation of apoptosis and deregulated gene expression. Consequently, all further defects reported would be secondary defects and not, as the authors claim, be a direct consequence of lack of Spastin.
The reviewer sees such an experiment as crucial to the interpretation of the data and the role Spastin.
Alternatively, the authors could re-organize the manuscript, focus on the primary defect during meiosis and describe all further effects as secondary.

Further points:
1) The Spast heterozygous males need to be mentioned in the initial analysis. Here, only Wt and ko animals are listed.
2) Further to that give information about female fertility.
3) Line 155 - the n-numbers given indicate males or plugs – please clarify
4) Line 158 – explain the method of “daily sperm production” since it is not clear how this was measured
5) General note to the arrowheads – improve the visibility by choosing better color scheme
6) Line 173 – Fig 2 D -please provide higher magnification to see this.
7) Line 181 – The reviewer sees significant increase in apoptosis, however, this seems to be in the range of +2% - thus the authors might consider mentioning this fact
8) The finding of misaligned chromosomes should be elaborated by an alternative method. A spindle staining and e.g. confocal imaging could further validate the findings.
9) Acrosome development – please provide a further method to demonstrate this. PNA-lectin staining on testes squash samples could be used.
10) Figure 6C is not mentioned in the text.
11) In order to clearly mark the manchette an appropriate antibody staining should be applied.
12) Line 428 – western blotting is mentioned, but no WB is shown.

Reviewer 2

Advance summary and potential significance to field

The authors performed phenotype analyses of Spastin-disrupted mice and unveiled that spastin is essential for proper male meiosis, acrosome formation, manchette structure, and nuclear integrity. Although the phenotypes of KO mice are interesting, this study lacks a mechanistic analysis of how spastin is involved in those phenotypes. Since spastin is a microtubule-severing protein it is interesting that it affects meiosis and manchette structure, which are closely related to microtubules. However, it is unfortunate that there is no analysis of why the abnormal severing of microtubules by spastin causes abnormalities in meiosis and the structure of the manchette.

© 2023. Published by The Company of Biologists under the terms of the Creative Commons Attribution License (https://creativecommons.org/licenses/by/4.0/).

Comments for the author

Since the phenotype of KO mice is interesting, we recommend that you do not further analyze it and submit it to a more specific journal. Alternatively, you may wish to resubmit it to Development once you have deciphered the mechanism.
Besides, the manuscript is generally verbose and contains information that is not so necessary (e.g., paragraphs 2-4 of the Introduction). The manuscript needs to be improved to make it more readable.

<u>Reviewer 3</u>

Advance summary and potential significance to field

This is the first description of the reproductive tract specific phenotype in the Spastic KO. The phenotype is

unique.

Comments for the author

This is a very nice publication and in general well-written and experiments of the highest quality. My suggestions for improvement are as follows:

1. Line 30-31: "supernumerary acrosome formation" should be replaced with the more broad phrase "acrosome biogenesis"

2. The Introduction appears somewhat long and the Discussion is more of a conclusion. Some aspects of the Intro could be moved to the discussion. In addition, it would be nice to add a figure 7 that summarizes phenotypes. There are some papers on acrosome biogenesis that have nice cartoons that could be modified to address the much more extensive phenotype for meiosis, manchette, and nuclear defects. This figure can then be part of the discussion. One question is whether the meiosis defects are causing many of the downstream defects (indirect) or whether spastin is directly involved in all of the activities; this is an important topic for the discussion.

3. Could the authors add a hit power image of some of the abnormal germ cells presented in Figure 4A?
Maybe such defects could be presented in Figure 7 instead of in Figure 4 and/or as a new figure in the supplement.

4. There are two panels in Figure 4 and 5 that are in common. The two panels under the acrosome phase are also presented as Figure 5B and 5K but flipped. Either the figure 4 panels or the figure 5 panels should be changed to be new images. If there are any other examples of similar use of the same images, those should be replaced.

First revision

<u>Author response to reviewers' comments</u>

<u>Response to Reviewers</u>
We thank the reviewers for the time and effort they have put into reviewing our manuscript. We appreciate their insights. We have addressed each comment below and, where possible, have added new data in response to the reviewers' comments. We note however, that only experiments that could make use of existing spastin knockout samples were possible, due to the loss of the spastin knockout mouse colony during the relocation of our lab to a new university. This aside, we believe that the additions made have addressed the concerns of the reviewers and significantly improved the manuscript. We trust that it is now acceptable for publication in *Development*.

© 2023. Published by The Company of Biologists under the terms of the Creative Commons Attribution License (https://creativecommons.org/licenses/by/4.0/).

Please note the line numbers detailed in our responses below refer to the version of the manuscript wherein changes are highlighted.

Reviewer 1

Advance Summary and Potential Significance to Field:
The paper by Cheers et al entitled “Spastin is an essential regulator of male meiosis, acrosome formation, manchette structure and nuclear integrity” describes the establishment and analysis of a Spastin-deficient mouse line. The authors report many pathophysiological changes and claim several to be intrinsically primary defects of loss of Spastin. While it is an interesting phenotype (which has been described in brief before by Tarrade et al. 2006) the authors need to address some points before the ms should be considered for publication.

Reviewer 1 Comments for the Author:
One major point is the lack of an appropriate antibody staining, which would clearly demonstrate the localization of Spastin during spermiogenesis. If Spastin can be detected in acrosome, manchette and nuclear membranes, that the argument, that the defects observed in ko animals are primary defects might hold. Without this, one might argue, that loss of Spastin leads to meiosis defects and, as a consequence to initiation of apoptosis and deregulated gene expression. Consequently, all further defects reported would be secondary defects and not, as the authors claim, be a direct consequence of lack of Spastin. The reviewer sees such an experiment as crucial to the interpretation of the data and the role Spastin. Alternatively, the authors could re-organize the manuscript, focus on the primary defect during meiosis and describe all further effects as secondary.

Response: We note that the report by Tarrade et al 2006 reports that mice are infertile only. No phenotypic or mechanistic data was provided in relation to fertility (Tarrade et al., 2006).

As requested, we have included additional data in the new Fig. S1 showing that spastin is localised in male germ cells during both meiosis and spermiogenesis using a validated antibody. We show in round spermatids spastin is localised to microtubules surrounding the developing acrosome and in elongating spermatids to the manchette (Fig. S1B). As noted in the text the hypothesis is that spastin regulates microtubules surrounding and within particular germ cell structures, rather than being an intrinsic component of the acrosome for example (lines 384-388). We also note that neither the acrosome nor manchette are present in meiotic cells thus meaning the chances of the defect being secondary to meiotic/spindle defects is minimal – *albeit* microtubules may be ‘recycled’. We accept however, that the nuclear fragility may be a consequence of a failure to disassemble spindle microtubules at the end of meiosis. This interpretation is included in lines 388-391. Single cell analyses of the testis RNA transcriptome have also previously shown that *Spast* is enriched in both spermatocytes and spermatids in humans and mice (Ernst et al., 2019, Wang et al., 2018, Chen et al., 2018, Guo et al., 2018). As such, to complement our immunolocalization data, we have included *Spast* expression profile data in Fig. S1C.

Further points:

1. The Spast heterozygous males need to be mentioned in the initial analysis. Here, only Wt and ko animals are listed.

   Response: We have updated the results text accordingly (lines 163-164).

2. Further to that give information about female fertility.

   Response: We have updated the results text accordingly (lines 163-164).

3. Line 155 - the n-numbers given indicate males or plugs – please clarify

   Response: The text has been updated to provide greater clarity (line 162).

4. Line 158 – explain the method of “daily sperm production” since it is not clear how this was measured

© 2023. Published by The Company of Biologists under the terms of the Creative Commons Attribution License (https://creativecommons.org/licenses/by/4.0/).

Response: Further explanation of testis daily sperm production and epididymal sperm content has been included (lines 543-550) as found in (Dunleavy et al., 2021).

5. General note to the arrowheads – improve the visibility by choosing better color scheme

   Response: As requested, the arrowhead colours have been altered and the size increased to improve the contrast against the background image.

6. Line 173 – Fig 2 D -please provide higher magnification to see this.

   Response: We have replaced Fig. 2D with a higher magnification of testis sections to increase the clarity of the phenotype.

7. Line 181 – The reviewer sees significant increase in apoptosis, however, this seems to be in the range of +2% - thus the authors might consider mentioning this fact
   Response: The fold increase in caspase positive germ cells per seminiferous tubule is 2.6. The text has been updated accordingly (line 198).

8. The finding of misaligned chromosomes should be elaborated by an alternative method. A spindle staining and e.g. confocal imaging could further validate the findings.

   Response: We have included additional data (Fig. S1) to confirm that spastin is present throughout spermatogenesis, including during meiosis, where our data suggests it acts at the major microtubule-based structure, the spindle. This fits with previous data in *Drosophila* that found a role for spastin for spindle regulation (Zhang et al., 2007). Due to the *Spast* knockout mouse no longer being available to the authors, the authors were unable to assess knockout spindle structure in purified knock out germ cells. Despite this limitation, our existing data shows a clear difference in chromosome misalignment on the testis sections presented in Fig. 3

9. Acrosome development – please provide a further method to demonstrate this. PNA-lectin staining on testes squash samples could be used.

   Response: Respectfully, the authors disagree that a further method is needed to confirm the acrosome phenotype. This phenotype has been clearly demonstrated using both electron microscopy and light microscopy techniques. We note that the PAS staining method used herein is commonly used to visualise the acrosome, and visualisation of the acrosome with PNA-lectin will not offer additional information. As per Reviewers 3's suggestion we have however included an additional panel in Fig. 4 (new panel B) to more clearly demonstrate the acrosome phenotypes.

10. Figure 6C is not mentioned in the text.

    Response: The text has been updated accordingly (lines 321-322).

11. In order to clearly mark the manchette an appropriate antibody staining should be applied.

    Response: An antibody against α-tubulin is the most appropriate marker, noting that the manchette is a microtubule-based structure. The results have been confirmed using staining for polyglutamylated tubulin (Fig. 6D) and electron microscopy.

12. Line 428 – western blotting is mentioned, but no WB is shown.

    Response: Apologies for this. The text has been updated to remove this mention of western blotting.

© 2023. Published by The Company of Biologists under the terms of the Creative Commons Attribution License (https://creativecommons.org/licenses/by/4.0/).

Reviewer 2
Advance Summary and Potential Significance to Field:
The authors performed phenotype analyses of Spastin-disrupted mice and unveiled that spastin is essential for proper male meiosis, acrosome formation, manchette structure, and nuclear integrity. Although the phenotypes of KO mice are interesting, this study lacks a mechanistic analysis of how spastin is involved in those phenotypes. Since spastin is a microtubule-severing protein, it is interesting that it affects meiosis and manchette structure, which are closely related to microtubules. However, it is unfortunate that there is no analysis of why the abnormal severing of microtubules by spastin causes abnormalities in meiosis and the structure of the manchette.

Reviewer 2 Comments for the Author:
Since the phenotype of KO mice is interesting, we recommend that you do not further analyze it and submit it to a more specific journal. Alternatively, you may wish to resubmit it to Development once you have deciphered the mechanism. Besides, the manuscript is generally verbose and contains information that is not so necessary (e.g., paragraphs 2-4 of the Introduction). The manuscript needs to be improved to make it more readable.

Response: We thank Reviewer 2 for their advice and comments, however, we respectfully disagree that this study lacks mechanistic data. The phenotypes within genetically modified mice are a direct indication of the processes within which individual genes/proteins play a role. We have defined a mechanistic role for spastin in each of male meiosis (spindle structure/function, midbody abscission, and nuclear envelope reformation), acrosome biogenesis, and manchette structure and disassembly. The general mechanism by which spastin severs microtubules is already well described based on cell line data (Lacroix et al., 2010, Vietri et al., 2015, Roll-Mecak and Vale, 2008). What is yet to be elucidated is where spastin acts within individual cell types and biological processes *in vivo* – hence this study.

This aside, in this revised manuscript, we have now included additional data identifying the spastin testis interactome via a mass spectrometry proteomic approach (Fig. S3 and Tables S1- 3). This data reveals that spastin interacts with key proteins involved in the cytoskeleton (ARPC4, SEPT2, NUDC, DCTN2) and in chromatin regulation (H1-2/ H1-3, and H2AZ1/H2AZ2).

We also note that as a major developmental process, the processes underpinning spermatogenesis and male fertility sit squarely within the scope of *Development*.

In response to Reviewer 2's further comments and to those of Reviewer 3, the length of the introduction has been reduced to increase legibility of the manuscript, including removing paragraph 2 from the introduction and by relocating information in paragraph 4 to the discussion. However, we feel that information in the other specified paragraphs is essential to understanding the current field of spastin-related research and the context of this study.

Reviewer 3
Advance Summary and Potential Significance to Field:
This is the first description of the reproductive tract specific phenotype in the Spastic KO. The phenotype is unique.

Reviewer 3 Comments for the Author:
This is a very nice publication and in general well-written and experiments of the highest quality. My suggestions for improvement are as follows:

1. Line 30-31: "supernumerary acrosome formation" should be replaced with the more broad phrase "acrosome biogenesis"

   Response: The text has been updated accordingly (line 30)

2a. The Introduction appears somewhat long and the Discussion is more of a conclusion. Some aspects of the Intro could be moved to the discussion.

   Response: In line with this comment and those of Reviewer 2, the introduction has been shortened, and the discussion altered (see the response to Reviewer 2 for full details).

© 2023. Published by The Company of Biologists under the terms of the Creative Commons Attribution License (https://creativecommons.org/licenses/by/4.0/).

2b. In addition, it would be nice to add a figure 7 that summarizes phenotypes. There are some papers on acrosome biogenesis that have nice cartoons that could be modified to address the much more extensive phenotype for meiosis, manchette, and nuclear defects. This figure can then be part of the discussion. One question is whether the meiosis defects are causing many of the downstream defects (indirect) or whether spastin is directly involved in all of the activities; this is an important topic for the discussion.

Response: As per this reviewer's suggestion, we have included an additional figure (Fig. 7), summarising the spermatogenesis functions defined for spastin herein.

In regard to the post-meiotic phenotypes in the *Spast* knockout mice, as detailed above in response to Reviewer 1's first comment, we strongly believe the acrosome and manchette phenotypes are primary effects. We have included additional data (new Fig. S1) showing spastin is expressed in male germ cells during both meiosis and spermiogenesis. As above, structures including the acrosome and the manchette are build *de novo* within haploid male germ cells.

3. Could the authors add a hit power image of some of the abnormal germ cells presented in Figure 4A? Maybe such defects could be presented in Figure 7 instead of in Figure 4 and/or as a new figure in the supplement.
Response: We have included an additional panel in Fig. 4 (new panel B) to address this comment. This panel shows high power images of acrosome development in *Spast*$^{WT/WT}$ and *Spast*$^{KO/KO}$ mice.

4. There are two panels in Figure 4 and 5 that are in common. The two panels under the acrosome phase are also presented as Figure 5B and 5K but flipped. Either the figure 4 panels or the figure 5 panels should be changed to be new images. If there are any other examples of similar use of the same images, those should be replaced.

Response: We apologise for this oversight. Duplicate panels in later figures have been replaced with appropriate images, and no further duplicate panels exist.

References

CHEN, Y., ZHENG, Y., GAO, Y., LIN, Z., YANG, S., WANG, T., WANG, Q., XIE, N., HUA, R., LIU, M., SHA, J., GRISWOLD, M. D., LI, J., TANG, F. & TONG, M. H. 2018. Single-cell RNA-seq uncovers dynamic processes and critical regulators in mouse spermatogenesis. *Cell Res,* 28, 879-896.

DUNLEAVY, J. E. M., O'CONNOR, A. E., OKUDA, H., MERRINER, D. J. & O'BRYAN, M. K. 2021. KATNB1 is a master regulator of multiple katanin enzymes in male meiosis and haploid germ cell development. *Development,* 148.

ERNST, C., ELING, N., MARTINEZ-JIMENEZ, C. P., MARIONI, J. C. & ODOM, D. T. 2019. Staged developmental mapping and X chromosome transcriptional dynamics during mouse spermatogenesis. *Nature Communications,* 10, 1251.

GUO, J., GROW, E. J., MLCOCHOVA, H., MAHER, G. J., LINDSKOG, C., NIE, X., GUO, Y., TAKEI, Y., YUN, J., CAI, L., KIM, R., CARRELL, D. T., GORIELY, A., HOTALING, J. M. & CAIRNS, B. R. 2018. The adult human testis transcriptional cell atlas. *Cell Res,* 28, 1141-1157.

LACROIX, B., VAN DIJK, J., GOLD, N. D., GUIZETTI, J., ALDRIAN-HERRADA, G., ROGOWSKI, K., GERLICH, D. W. & JANKE, C. 2010. Tubulin polyglutamylation stimulates spastin-mediated microtubule severing. *J Cell Biol,* 189, 945-54.

ROLL-MECAK, A. & VALE, R. D. 2008. Structural basis of microtubule severing by the hereditary spastic paraplegia protein spastin. *Nature,* 451, 363-7.

TARRADE, A., FASSIER, C., COURAGEOT, S., CHARVIN, D., VITTE, J., PERIS, L., THOREL, A., MOUISEL, E., FONKNECHTEN, N., ROBLOT, N., SEILHEAN, D., DIERICH, A., HAUW, J. J. & MELKI, J. 2006. A mutation of spastin is responsible for swellings and impairment of transport in a region of axon characterized by changes in microtubule composition. *Hum Mol Genet,* 15, 3544-58.

VIETRI, M., SCHINK, K. O., CAMPSTEIJN, C., WEGNER, C. S., SCHULTZ, S. W., CHRIST, L., THORESEN, S. B., BRECH, A., RAIBORG, C. & STENMARK, H. 2015. Spastin and ESCRT-III

© 2023. Published by The Company of Biologists under the terms of the Creative Commons Attribution License (https://creativecommons.org/licenses/by/4.0/).

coordinate mitotic spindle disassembly and nuclear envelope sealing. *Nature,* 522, 231-5.
WANG, M., LIU, X., CHANG, G., CHEN, Y., AN, G., YAN, L., GAO, S., XU, Y., CUI, Y., DONG, J., CHEN, Y., FAN, X., HU, Y., SONG, K., ZHU, X., GAO, Y., YAO, Z., BIAN, S., HOU, Y., LU, J., WANG, R., FAN, Y., LIAN, Y., TANG, W., WANG, Y., LIU, J., ZHAO, L., WANG, L., LIU, Z., YUAN, R., SHI, Y., HU, B., REN, X., TANG, F., ZHAO, X. Y. & QIAO, J. 2018. Single-Cell RNA Sequencing Analysis Reveals Sequential Cell Fate Transition during Human Spermatogenesis. *Cell Stem Cell,* 23, 599-614 e4.
ZHANG, D., ROGERS, G. C., BUSTER, D. W. & SHARP, D. J. 2007. Three microtubule severing enzymes contribute to the "Pacman-flux" machinery that moves chromosomes. *Journal of Cell Biology,* 177, 231-242.

Second decision letter

MS ID#: DEVELOP/2022/201183

MS TITLE: Spastin is an essential regulator of male meiosis, acrosome formation, manchette structure and nuclear integrity

AUTHORS: Samuel R Cheers, Anne E O'Connor, Travis K Johnson, D. Jo Merriner, Moira K O'Bryan, and Jessica EM Dunleavy

I have now received all the referees' reports on the above manuscript, and have reached a decision. The referees' comments are appended below, or you can access them online: please go to BenchPress and click on the 'Manuscripts with Decisions' queue in the Author Area.

As you will see, the referees found the manuscript considerably improved. However, reviewer 2 still expressed some significant criticisms and recommend a substantial revision of your manuscript before we can consider publication. If you are able to revise the manuscript along the lines suggested, which may involve further experiments, I will be happy receive a revised version of the manuscript. Your revised paper will be re-reviewed by one or more of the original referees, and acceptance of your manuscript will depend on your addressing satisfactorily the reviewers' major concerns. Please also note that Development will normally permit only one round of major revision. If it would be helpful, you are welcome to contact us to discuss your revision in greater detail. Please send us a point-by-point response indicating your plans for addressing the referee’s comments, and we will look over this and provide further guidance.

Please attend to all of the reviewers' comments and ensure that you clearly highlight all changes made in the revised manuscript. Please avoid using 'Tracked changes' in Word files as these are lost in PDF conversion. I should be grateful if you would also provide a point-by-point response detailing how you have dealt with the points raised by the reviewers in the 'Response to Reviewers' box. If you do not agree with any of their criticisms or suggestions please explain clearly why this is so.

Reviewer 1

Advance summary and potential significance to field

All my points have been addressed. No further comments.

Comments for the author

All my points have been addressed. No further comments.

Reviewer 2

Advance summary and potential significance to field

© 2023. Published by The Company of Biologists under the terms of the Creative Commons Attribution License (https://creativecommons.org/licenses/by/4.0/).

In this revision, additional experiments were performed to examine spastin localization and to identify spastin-interacting proteins by MS analysis. Thus the content has been improved compared to the previous submission. However, the results section contains lengthy descriptions of prior literature other than the experimental results and overstatements.

Comments for the author

Major points
The results of Fig S1B are very important for understanding the function of spastin in germ cells. However, the data in Fig S1A, which examined the localization of spastin in the testis, shows that spastin is located far from the nucleus, which may be quite different from the data in Fig S1B. Please reconfirm. Also, I would like an updated, partially enlarged figure, as the current figure does not show enough detail. I would also like to see DAPI-only images in Fig S1B. Finally, you say there is a difference in mRNA expression in Fig 1D, but why is there no difference in Spastin expression in the WB analysis?

It is gratifying that the MS analysis revealed that factors associated with spastin in previous literature also interact in the testis. However, whether the phenotype emerged directly due to the interaction between Spastin and these factors is questionable. It would help to understand how Spastin functions if you clarified the changes in protein localization and expression of NUDC, DCTN2 and SEPT2.

KO mice are deficient in spastin throughout the body, but only meiosis seems to be affected (although you have not observed somatic cell division). Despite this, is it correct to analogize the function of spastin by citing the data seen in somatic cell division? The authors also need to consider why somatic cell division was not affected in spastin KO mice.

Please correct the order of the figures to the order in the manuscript.

In the discussion section, many contents that are far removed from the experimental results are described.

Minor points
Line 126-127 Are the KO mice displaying any abnormalities in their appearance such as spastic paralgesia?

Line133 To clarify whether the mating behavior is normal, please describe the plug numbers of the KO male. In addition, please define the term fertility test in the “materials and methods.”

Fig. 1A I’m confused by the diagram. Was this cassette inserted into intron 4 of Spatin4 to make Spat4 KO mice?-What is the meaning of the dotted line in Fig. 1A? Could you add the diagram for the KO allele in the KO mice? In addition, I think Fig. 1A and 1B are too close (Fig. 1B and 1C too).

Line 199 Why don’t you explain Fig. 3C here? The TEM figure makes it easy to understand the existence of binucleated spermatids.

Line 234-237 “A similar compacted acrosome phenotype was seen in the spermatids from Spast KO/KO males, however, multiple acrosome compartments were still observed (Fig. 4C, red arrowheads), in addition to a loss of nuclear membrane integrity (Fig. 4C, green arrowhead).” Please check this sentence. There are several wrong statements.

Line 237-240 “Electron microscopy revealed that docking of acrosomal vesicles throughout development, starting in the Golgi phase, was associated with an abnormally deep nuclear membrane invagination in spermatids from SpastKO/KO males, suggesting compromised nuclear integrity (Fig. 4C, blue arrowheads).” I think this is this sentence is for Fig. 4D
Line 323-363 These sentences are too redundant, although these are not experimental results.

Line337-347 IP-MS analysis only revealed the interactions between SPAST and SEPT2/Histone proteins. These sentences include over-interpretation. To write these sentences you must perform additional experiments.

© 2023. Published by The Company of Biologists under the terms of the Creative Commons Attribution License (https://creativecommons.org/licenses/by/4.0/).

Line 367 “While no defects were apparent during SpastKO/KO male germ cell mitosis,” Did you confirm that mitosis is normal in the KO male?

Second revision

Author response to reviewers' comments

Response to Reviewers
We thank the reviewers for the time and effort they have put into reviewing the revised version of our manuscript. We have addressed the comments below and made changes to increase the clarity of our work. We note however that some of the suggested changes are beyond the scope of the study and well beyond the scope of the revisions requested. We believe the alterations we have made should address the concerns of the reviewers and trust that the manuscript is now acceptable for publication in *Development*.

Please note the line numbers detailed in our responses below refer to the version of the manuscript wherein changes are highlighted.

Reviewer 1
Advance Summary and Potential Significance to Field:
All my points have been addressed. No further comments.

Reviewer 1 Comments for the Author:
All my points have been addressed. No further comments.

Reviewer 2
Advance Summary and Potential Significance to Field:
In this revision, additional experiments were performed to examine spastin localization and to identify spastin-interacting proteins by MS analysis. Thus, the content has been improved compared to the previous submission. However, the results section contains lengthy descriptions of prior literature other than the experimental results and overstatements.

Reviewer 2 Comments for the Author:
Major points
The results of Fig S1B are very important for understanding the function of spastin in germ cells. However, the data in Fig S1A, which examined the localization of spastin in the testis, shows that spastin is located far from the nucleus, which may be quite different from the data in Fig S1B. Please reconfirm. Also, I would like an updated, partially enlarged figure, as the current figure does not show enough detail. I would also like to see DAPI-only images in Fig S1B. Finally, you say there is a difference in mRNA expression in Fig 1D, but why is there no difference in Spastin expression in the WB analysis?

Response: As requested, a DAPI only panel for Fig S1B (now Fig S1C) and image panels of increased magnification have been added for Fig S1A (Fig S1B). In respect to the differences in localisation observed between panel S1A and S1B (now S1C), Fig S1A depicts testis sections, and Fig S1B (now S1C) depicts isolated germ cells, which allow for more precise localisation. Fig S1A only serves to validate the antibody in wild-type versus knockout tissues (noting due to the loss of our knockout tissue Bouin’s fixed paraffin testis sections were all we had available). Whereas to define its sub-cellular localisation wildtype isolated germ cells were used in Fig S1B (now S1C). We are unsure what western blot the reviewer is referring to. A western blot was not performed as it was deemed not essential following the significant decrease of mRNA seen in the qPCR.

It is gratifying that the MS analysis revealed that factors associated with spastin in previous literature also interact in the testis. However, whether the phenotype emerged directly due to the interaction between Spastin and these factors is questionable. It would help to understand how Spastin functions if you clarified the changes in protein localization and expression of NUDC, DCTN2, and SEPT2.

© 2023. Published by The Company of Biologists under the terms of the Creative Commons Attribution License (https://creativecommons.org/licenses/by/4.0/).

Response: While we appreciate the reviewer's interest, the localisation of the binding partners is beyond the scope of this study – which was to test the requirement for SPAST in male fertility. In addition, we have not claimed that the mislocalization of these proteins is the only cause of the phenotype. As described, the cause of infertility is failure of meiosis followed by a catastrophic loss of nuclear integrity.

KO mice are deficient in spastin throughout the body, but only meiosis seems to be affected (although you have not observed somatic cell division). Despite this, is it correct to analogize the function of spastin by citing the data seen in somatic cell division? The authors also need to consider why somatic cell division was not affected in spastin KO mice.

Response: In addition to meiosis defects, the loss of SPAST results in severe defects in haploid germ cell development. As interpreted by the reviewer, SPAST is not essential for mitosis as evidenced by whole body weight. Mitosis and meiosis are distinct processes with different regulation, though similarities do exist. It is reasonable to conclude that another severing enzyme is capable of compensating for the loss of spastin during mitosis, as stated at lines 381-382. The compromise of nuclear integrity caused by the loss of spastin during mitosis noted by (Vietri et al., 2015) was identified *in vitro* in HeLa cells. Two possibilities exist as to why we observed this defect in meiosis and not in mitosis; first, there is a compensatory mechanism in the mouse that is absent *in vitro* or in human cells, second, it is the subsequent morphological changes that comprise spermiogenesis acting upon the compromised nucleus that cause catastrophic failure of nuclear integrity.

Please correct the order of the figures to the order in the manuscript.

Response: With respect, as far as the authors can tell the figures are in the correct order in the manuscript.

In the discussion section, many contents that are far removed from the experimental results are described.

Response: We are unable to address this comment in the absence of specific examples.

<u>Minor points</u>
Line 126-127 Are the KO mice displaying any abnormalities in their appearance, such as spastic paralgesia?

Response: As stated in the manuscript on lines 129, the mice did not show any overt abnormalities. Given that we assess fertility in mice at approximately 3 months of age, the failure to see a spasticity phenotype is not unexpected. Previous assessments with a similar spastin knockout mouse model that focused on behaviour and the brain found that mice displayed a progressive spasticity phenotype wherein mild motor deficits were observed in mice aged to 22-24 months of age, but were not observed in the younger times points of 4, 12 and 15 months of age (Tarrade et al., 2006).

Line 133 To clarify whether the mating behavior is normal, please describe the plug numbers of the KO male. In addition, please define the term fertility test in the "materials and methods."

Response: The text has been updated to reflect these requests. At line 132 the number of copulatory plugs has been included. At line 506, the fertility test is already explained in full.

Fig. 1A I'm confused by the diagram. Was this cassette inserted into intron 4 of Spatin4 to make Spat4 KO mice? What is the meaning of the dotted line in Fig. 1A? Could you add the diagram for the KO allele in the KO mice? In addition, I think Fig. 1A and 1B are too close (Fig. 1B and 1C too).
Response: Yes, this diagram indicates that the cassette was inserted into intron 4. This is the standard diagram provided by the trans-NIH Knockout Mouse Project where the allele was sourced from and therefore, we are unable to alter it. The dotted line indicates that splicing has occurred between exon 4 and the construct, allowing for the generation of the knockout. We have clarified the wording of the figure legend. We have increased the spacing between each panel as requested.

© 2023. Published by The Company of Biologists under the terms of the Creative Commons Attribution License (https://creativecommons.org/licenses/by/4.0/).

Line 199 Why don’t you explain Fig. 3C here? The TEM figure makes it easy to understand the existence of binucleated spermatids.

Response: Figure 3C does not depict a binucleated spermatid, it depicts a single nucleus traversing an intercellular bridge. This is a similar but distinct phenotype and it would not be appropriate to discuss in the position indicated.

Line 234-237 “A similar compacted acrosome phenotype was seen in the spermatids from Spast KO/KO males, however, multiple acrosome compartments were still observed (Fig. 4C, red arrowheads), in addition to a loss of nuclear membrane integrity (Fig. 4C, green arrowhead).” Please check this sentence. There are several wrong statements.

Response: The authors apologise for the confusion, the statements made are correct but the arrowhead colours were changed in response to the last revision. The references to the figure have been amended to correctly reflect what is shown.

Line 237-240 “Electron microscopy revealed that docking of acrosomal vesicles throughout development, starting in the Golgi phase, was associated with an abnormally deep nuclear membrane invagination in spermatids from SpastKO/KO males, suggesting compromised nuclear integrity (Fig. 4C, blue arrowheads).” I think this is this sentence is for Fig. 4D.

Response: This sentence refers to processes being shown in both Fig. 4C and Fig 4D and has been updated to include references to both figures

Line 323-363 These sentences are too redundant, although these are not experimental results.

Response: With respect, at the indicated position the authors detect no redundancy in the explanation of the importance of the dynein-dynactin complex.

Line337-347
IP-MS analysis only revealed the interactions between SPAST and SEPT2/Histone proteins. These sentences include over-interpretation. To write these sentences, you must perform additional experiments.

Response: Respectfully, we disagree that we over-interpret at this position. We state previously known data regarding the severing of poly-glutamylated microtubules by spastin and that SEPT2 relies on poly-glutamylated microtubules to perform a relevant function. We then theorise that the loss of spastin mediated microtubule severing could result in a disruption to the function of SEPT2. Regarding the histone proteins, we only note that spastin has interactions with these and that it could be relevant to the nuclear phenotype we observed.

Line 367 “While no defects were apparent during SpastKO/KO male germ cell mitosis,” Did you confirm that mitosis is normal in the KO male?

Response: This has been explored through an overall assessment of health using body weight and directly observed and shown in the caspase staining present in Fig S2.

TARRADE, A., FASSIER, C., COURAGEOT, S., CHARVIN, D., VITTE, J., PERIS, L., THOREL, A., MOUISEL, E., FONKNECHTEN, N., ROBLOT, N., SEILHEAN, D., DIERICH, A., HAUW, J. J. & MELKI, J. 2006. A mutaBon of spasBn is responsible for swellings and impairment of transport in a region of axon characterized by changes in microtubule composiBon. *Hum Mol Genet,* 15, 3544-58.

VIETRI, M., SCHINK, K. O., CAMPSTEIJN, C., WEGNER, C. S., SCHULTZ, S. W., CHRIST, L., THORESEN, S. B., BRECH, A., RAIBORG, C. & STENMARK, H. 2015. SpasBn and ESCRT-III coordinate mitoBc spindle disassembly and nuclear envelope sealing. *Nature,* 522, 231-5.

© 2023. Published by The Company of Biologists under the terms of the Creative Commons Attribution License (https://creativecommons.org/licenses/by/4.0/).

Third decision letter

MS ID#: DEVELOP/2022/201183

MS TITLE: Spastin is an essential regulator of male meiosis, acrosome formation, manchette structure and nuclear integrity

AUTHORS: Samuel R Cheers, Anne E O'Connor, Travis K Johnson, D. Jo Merriner, Moira K O'Bryan, and Jessica EM Dunleavy
ARTICLE TYPE: Research Article

I am happy to tell you that your manuscript has been accepted for publication in Development, pending our standard ethics checks.

© 2023. Published by The Company of Biologists under the terms of the Creative Commons Attribution License (https://creativecommons.org/licenses/by/4.0/).
